# Non-Homologous End Joining and Homology Directed DNA Repair Frequency of Double-Stranded Breaks Introduced by Genome Editing Reagents

**DOI:** 10.1371/journal.pone.0169931

**Published:** 2017-01-17

**Authors:** Michail Zaboikin, Tatiana Zaboikina, Carl Freter, Narasimhachar Srinivasakumar

**Affiliations:** Division of Hematology/Oncology, Department of Internal Medicine, Saint Louis University, Saint Louis, Missouri, United States of America; Temple University School of Medicine, UNITED STATES

## Abstract

Genome editing using transcription-activator like effector nucleases or RNA guided nucleases allows one to precisely engineer desired changes within a given target sequence. The genome editing reagents introduce double stranded breaks (DSBs) at the target site which can then undergo DNA repair by non-homologous end joining (NHEJ) or homology directed recombination (HDR) when a template DNA molecule is available. NHEJ repair results in indel mutations at the target site. As PCR amplified products from mutant target regions are likely to exhibit different melting profiles than PCR products amplified from wild type target region, we designed a high resolution melting analysis (HRMA) for rapid identification of efficient genome editing reagents. We also designed TaqMan assays using probes situated across the cut site to discriminate wild type from mutant sequences present after genome editing. The experiments revealed that the sensitivity of the assays to detect NHEJ-mediated DNA repair could be enhanced by selection of transfected cells to reduce the contribution of unmodified genomic DNA from untransfected cells to the DNA melting profile. The presence of donor template DNA lacking the target sequence at the time of genome editing further enhanced the sensitivity of the assays for detection of mutant DNA molecules by excluding the wild-type sequences modified by HDR. A second TaqMan probe that bound to an adjacent site, outside of the primary target cut site, was used to directly determine the contribution of HDR to DNA repair in the presence of the donor template sequence. The TaqMan qPCR assay, designed to measure the contribution of NHEJ and HDR in DNA repair, corroborated the results from HRMA. The data indicated that genome editing reagents can produce DSBs at high efficiency in HEK293T cells but a significant proportion of these are likely masked by reversion to wild type as a result of HDR. Supplying a donor plasmid to provide a template for HDR (that eliminates a PCR amplifiable target) revealed these cryptic DSBs and facilitated the determination of the true efficacy of genome editing reagents. The results indicated that in HEK293T cells, approximately 40% of the DSBs introduced by genome editing, were available for participation in HDR.

## Introduction

Genome editing has gained popularity due to the ease of designing sequence-specific endonucleases based on either transcription-activator like effector nucleases (TALENs) [1] or RNA guided endonucleases (RGENs)[2].

TALENs are derivatives of transcription-activator like effector (TALE) proteins that are produced by plant pathogens belonging to Xanthomonas sps that subvert gene expression in plant cells for their own benefit [3]. These proteins recognize DNA sequences by virtue of 32–33 amino acid repeats. Each repeat has a pair of residues (Repeat Variable Di-residue) which confers the specificity of its binding to a particular base. By combining repeats with the right specificities it is possible to engineer a TALE protein to bind to any sequence of interest. Libraries of individual and combinations of repeats are available to facilitate the rapid construction of TALEs of desired specificity[4]. In frame fusion of the TALE protein to an endonuclease such as FokI results in a TALEN that functions only as a dimer. Two TALENs targeting opposite strands of DNA and spaced appropriately are required to activate the FokI nuclease by dimerization to effect a double-stranded break (DSB) in the target locus[1,5,6]. These stringent requirements ensure that properly designed TALEN pairs are specific with low probability of off-target effects.

CRISPR stands for clustered regularly interspaced short palindromic repeats. In combination with a CRISPR associated endonuclease (Cas9), the CRISPR/Cas9 system constitutes a bacterial adaptive immune mechanism that protects the bacterium from foreign genetic elements including bacteriophages [7]. The specificity of this system derives from expressed short CRISPR RNAs (crRNAs) that contain ~20 bp sequence complementary to the target sequence on the foreign DNA. A common transactivating RNA (trRNA) recruits the Cas9 endonuclease to effect a DSB on the target DNA. This system has been adapted to function in mammalian cells. Many clever modifications have been made to this system to improve its specificity and decrease off-target effects. One such modification consists of using an RNA that contains both target recognition sequence performed by crRNA and the Cas9 tethering function of trRNA as a single guide RNA molecule (sgRNA or gRNA) [8]. Other modifications include the use of a fusion protein consisting of a noncleaving mutant Cas9 to FokI endonuclease (dCas9-FokI) such that DSBs are only produced when two gRNAs are targeted to two distinct sequences (~20 bases long) on opposite strands of the target DNA, and spaced appropriately (~16 bases) for the dCas9-FokI to dimerize and initiate the DSB [9]. This system is also referred to as RNA guided Fok1 nuclease (RFN) system. Recent versions of this system use truncated gRNAs to improve the specificity of RFNs as RFNs have been previously shown to produce a low frequency of indels at the target site with even a single gRNA [10].

The DSBs effected by genome editing reagents in eukaryotic cells undergo repair using either non-homologous end joining (NHEJ) or homology-directed repair (HDR) pathways [11–13] Although NHEJ repair is believed to occur throughout the cell cycle, it predominantly occurs during G1 phase. Many of the enzymes involved in NHEJ (XRCC6, PARP3, PNKP, CRCC4, NHEJ1) with few exceptions (e.g., XRCC5 and PRKDC in G1, LIG4 and PCNA in S) do not exhibit a cell cycle specific expression pattern [12–14]. In contrast, HDR is most prominent during S and G2 phases [15–17] These differences are thought to be due to the expression of enzymes involved in HDR in a cell cycle dependent manner. For example, RAD50 and MRE11A within the MRN complex, BRCA1, EXO1, BLM, DNA2, RAD51, RAD54B, POLD1, POLD3, PCNA, etc are all predominantly expressed in S phase and involved in HDR. During S/early G2 phases, the sister chromatid can serve as template for repair. It has been suggested that the absence of a template tilts the repair mechanisms to favor NHEJ [15–18].

Real-time PCR that use non-specific DNA binding dyes allows one to determine the melting profile of PCR products immediately after completion of amplification of target region. The dyes bind to double-stranded DNA and emit fluorescence upon excitation at the appropriate wavelength. The DNA melting profile is generated by gradually increasing the temperature in small increments during which the double-stranded DNA binding dye is progressively expelled as regions of low TM become single-stranded resulting in a decrease in fluorescence intensity [19]. The melting profile is dependent on the GC content, length and sequence. The temperature at which half of the DNA molecules are single-stranded is defined as the melting temperature or Tm. High resolution melting analysis (HRMA) has been previously used to distinguish homozygous and heterozygous alleles in target genomic DNA (gDNA) [19, 20]. The DSBs at the target sites after NHEJ repair resulting in indels should also enable the detection of presence of such molecules by HRMA[21–23]. The detection efficiency is expected to depend on the efficiency of genome editing reagents. Since HRMA is biased towards detection of target sites undergoing NHEJ repair, we also investigated the effect of including a donor template on NHEJ repair at sites targeted by genome editing reagents. The results indicate that HRMA can be used as a quantitative tool to identify the efficacy of genome editing reagents. The presence of a donor template during genome editing enhanced the sensitivity of the detection of mutant target sites by HRMA by removal of wild type target sequence amplifiable by the PCR primers used in HRMA.

An alternative approach to discriminate mutant from wild type target sequence is to use TaqMan probes that bind over the target cut sites of genome editing reagents. The proportion of target sequence modified by either NHEJ or HDR in the presence of donor template was deduced from the reduction in binding of the TaqMan probe and consequent diminution in qPCR fluorescent signal. The results of both HRMA and TaqMan assays were complementary and indicated that approximately 40% of DSBs were amenable for HDR in the presence of donor template in HEK293T cells.

## Materials and Methods

### Cells

Human embryonic kidney 293T (HEK293T) cells were obtained from American Type Culture Collection (ATTC; catalog number SD-3515). The cells were maintained in Dulbecco’s modified Eagle’s medium containing 2 mM L-glutamine, 100 U/ml of penicillin, 100 μg/ml streptomycin and 10% heat-inactivated fetal bovine serum (FBS) (Hyclone/ThermoFisherScientific, USA). Human lung fibroblasts (MRC-5) were obtained from ATCC (catalog number CCL-171) and maintained in Eagle’s minimal essential medium containing 10% FBS.

### Plasmids

#### Design and construction of TALENs targeting CCR5 locus

The target sequence derived from c-c motif chemokine receptor 5 (CCR5, GenBank RefSeqGene number NG_012637), nt 8761 to nt 8880, encompassing the 3’ end of coding region (exon 3) and the downstream non-coding intron region was used in the ZiFit Targeter online tool (http://zifit.partners.org/ZiFiT/TALZiFiTNuclease.aspx) to design the TALENs. Several pairs of putative TALENS were identified in the output of the online tool that effected a cleavage at position 8837 in the down-stream noncoding region of the CCR5. The TALEN kit used for TALE assembly was a gift from Keith Joung (Addgene kit # 1000000017). The protocol accompanying the TALEN Assembly Kit was used for construction of TALENs. The scheme of assembly of the right and left TALENs is shown in S1 Fig. The sequences recognized by the right and left TALENs used in this study, and the spacer sequence between the TALENs are shown in Table 1.

**Table 1:** Target sequences in the CCR5 locus for TALENs and the F8 locus for RGEN.

Target^a^	GenBank Accession Number	Target sequence^b^ (5’-3’)
CCR5 intron	NG_012637	TGGGCTTGTGACACGGACtcaagtggg**c**tggtgaCCCAGTCAGAGTTGTGCA
F8 intron 1-S1	NG_011403	CCAGGATTGTGGGGATGTAAGTctgcttggaggaaggTGCAGACATCGGGTTAGGATGG
F8 intron 1-S2	NG_011403	CCATTGCTTCTCTTCGTCATATGctgctcctccagaatcTAGAGACTGGAGTAGAGGGAGGG
F8 intron 1-S3	NG_011403	CCTACTTTGGGTGTAAGGATAATatgagccttgagttcAGAAGCTTTTCGTGTTTTGGGGG

^a^S1, S2 and S3 refer to three neighboring dimeric gRNA target sites in intron 1 of human coagulation factor VIII (F8).

^b^The spacer sequences are shown in lower case while the left and the right recognition sequences for CCR5 TALENs or for F8 RGENs are shown in upper case. The underlined ‘C’ in the CCR5 target sequence was identified as the desired cleavage site for ZiFit Targeter online design tool.

#### Design and construction of gRNA encoding constructs targeting intron 1 of human coagulation factor VIII (F8)

We used the dimeric gRNA-guided FokI nuclease system described by the Joung Lab for targeting human coagulation factor VIII (F8) locus[9]. The system consists of the plasmid pSQT1313 and pSQT1601. pSQT1313 and pSQT1601 were gifts from Keith Joung (Addgene plasmid # 53370 and # 53369, respectively). The plasmid pSQT1313 allows the simultaneous expression of both the left and right gRNAs under control of the U6 promoter. The two gRNAs are derived from a single transcript by virtue of the presence of cleavage sites for *Pseudomonas aeruginosa* Csy4 RNase. The expression construct pSQT1601 encodes Csy4 RNase as part of a fusion protein with dCas9-Fok1 and separated by a self-cleaving T2A protease. Three different target sites within intron 1 of the F8 locus (F8-S1, F8-S2, and F8-S3, Table 1) were identified using the online tool (ZiFit Targeter). The left and right gRNAs for each of the target sites were cloned into pSQT1313 by separately annealing and assembling the left, middle and right oligoduplexes (S1 Table) and combining them prior to ligation into pSQ1313 that was prepared by cleavage with BsmB1. An example of the cloning strategy and the final construct is shown in S2 Fig. The resulting clones, pSQT1313_F8S1, pSQT1313_F8S2, pSQT1313_F8S3, were verified by restriction enzyme analysis using NdeI and Sanger sequencing. The pSQT1313_F8S2 clone 11 showed a single base deletion within the middle oligo sequence (S1 Table, ‘C’ nucleotide shown in red).

#### Plasmid for providing donor template to effect HDR

The donor plasmids for providing the template for HDR at the target DSB site were created using the plasmid vector HR110PA-1 (System Biosciences, CA, USA). We refer to this plasmid henceforth as pBackbone. Plasmid pBackbone contains an expression cassette that encodes for a fusion protein consisting of a red fluorescent protein (RFP) and puromycin resistance marker under control of the elongation factor 1 alpha promoter. This expression cassette is situated between multiple cloning sites for the left and right homology arms necessary for effecting HDR.

#### Donor plasmid for homologous recombination at the CCR5 locus

The primer sequences used for creating the template donor construct are shown in S2 Table. The right homology arm (CCR5_RHA) was amplified from human genomic DNA isolated from MRC5 cells using primers SK164 and SK165 that also introduced BamHI and SphI restriction enzyme digestion sites, respectively. These primers amplified a region between nt 8898 and nt 9855 of CCR5 RefSeqGene (GenBank Accession Number NG_012637). The amplified PCR product was digested with BamHI and SphI and inserted into pBackbone cut with the same restriction enzymes. The wild-type left homology arm (CCR5_LHA) was amplified from human genomic DNA using primers SK162 and SK163 that amplify the region between nt 7415 and nt 8366 of CCR5 RefSeqGene, and inserted into the BglII of pBackbone via ligation-independent cloning to create the CCR5 plasmid pSK787 (HR-LHA-2-RHA). A cassette encoding enhanced green fluorescent protein (EGFP) under control of the HIV-1 long terminal repeat (LTR) promoter and terminated by bovine growth hormone polyA (bGHpA) signal fragment was derived by splicing by overlap extension PCR using primers SK169 and SK170 for amplifying 3’ LTR of HIV-1 molecular clone pNL4-3 (corresponding to nt 9076 to nt 9709 of GenBank Accession number M19921.2) in plasmid construct pBS-3’LTR (unpublished), SK171 and SK172 (for amplifying EGFP from pEGFP-N1 (Clontech, USA) corresponding to nt 679 to nt 1398), SK173 and SK174 (for amplifying bGHpA corresponding to nt 1042 to nt 1249 of pCDNA3 (Invitrogen, USA) in plasmid construct pBLUΔ-4pA (unpublished), and inserted downstream of the LHA by ligation-independent closing at the XbaI site to obtain the donor plasmid pDonor-CCR5 (pSK791 /HR-LHA-2-RHA-LGpA). A flow diagram depicting the cloning steps is shown in S3 Fig. A plasmid map exhibiting the main features is shown in S5A Fig.

#### Donor plasmid targeting F8 intron 1 (pDonor-F8)

The F8 donor plasmid was created as follows: The sequence between nt 5594 and nt 6430 in RefSeqGene NG_011403 was amplified from human genomic DNA using primers containing BamHI and SphI sites in the sense and antisense primers (S3 Table), respectively, and inserted into pBackbone cut with the same enzymes to introduce the right homology arm (853 bp). To generate the left homology arm, genomic DNA, corresponding to nt 4582 and 5300 of RefSeqGene NG_011403, was amplified using forward primer containing the Acc65I restriction enzyme site. This fragment was then fused in frame by splicing overlap extension (SOE)-PCR to a fragment containing EGFP followed by bovine growth hormone poly A signal. An NsiI site was introduced at the 3’ end during SOE-PCR. The final product was digested with Acc65I and NsiI and cloned into the pBackbone containing the right homology arm cut with the same restriction enzymes to generate the plasmid pDonor-F8 (HR-F8LHA_GpA-RHA). The primers sequences used in this cloning are shown in S3 Table, the cloning steps are outlined in S4 Fig, and a plasmid map showing the main features is shown in S5B Fig.

#### Plasmid maps

Plasmid maps and *in silico* cloning were done using SnapGene v2.8.3 software (GSL Biotech LLC, Chicago, IL, USA).

### Transfection and selection of HEK293T cells

The HEK293T cells were seeded in 6-well plates (0.75 x 10^6^ cells/well) one day prior to transfection to achieve a cell density of approximately 60–80% at the time of transfection. Plasmid DNA was introduced into the cells by CaPO_4_-mediated transient transfection protocol as described previously [24]. Briefly, plasmid DNA (≤ 5 μg total) was resuspended in 0.25 ml of CaCl_2_ solution (0.25 M) in a 2 ml microcentrifuge tube. The DNA was precipitated by adding 0.25 ml of HEPES buffered saline, pH 7.05 (50 mM HEPES, 10 mM KCl, 12 mM dextrose, 280 mM NaCl, 1.5 mM Na_2_HPO_4_) drop-wise, while bubbling air through the DNA-CaCl_2_ solution using a sterile one-ml plastic pipet. The solution was promptly distributed onto the media over the cells in each well. The medium was replaced with fresh medium the following day. The next day, wells were washed twice with 1 ml of phosphate buffered saline and the attached cells were released by treatment with 0.3ml of trypsin (0.25%)-EDTA (1 mM). The trypsin-EDTA was neutralized by addition of 0.9 ml of complete medium. The cells were either used for isolation of gDNA or placed in puromycin-containing medium (2 μg/ml). The medium was replaced twice weekly for about 2 weeks when colonies were discernible in test wells but none were seen in control wells containing untransfected control HEK293T cells. The gDNA was isolated from pools of puromycin-resistant colonies using a Qiagen DNeasy Blood and Tissue Kit ((Maryland, USA, catalog number 69504) as per the manufacturer’s recommended protocol. Contaminating RNA was removed by including an RNase A treatment step during the lysis step as recommended by the manufacturer.

### HRMA

The target regions in CCR5 or F8 intron 1 containing the cleavage sites for TALENs or RGENs were amplified using appropriate oligonucleotide primers (Table 2) in a Bio-Rad CFX96 real-time PCR machine using the Precision Melt Supermix buffer (Bio-Rad, USA). The following cycling parameters were used: An initial denaturation cycle at 98°C for 2 min, 40 cycles of amplification consisting of a denaturation step at 98°C for 5s and an annealing/extension step at 59.5°C for 10s, followed by a cycle at 95°C for 1 min, a cycle at 55°C for 1 min and a melt curve setting of 70°C to 95°C with 0.2°C increments and 10s/step. The data were exported and analyzed in Excel or Numbers software programs (Mac Os X) as follows: From the melting profile, the low and high temperature limits or window for 100% and 0% fluorescence were determined. The baseline fluorescence at the cut off temperature was subtracted from melting profile for each curve to provide the 0% fluorescence value. Then the melting profile was normalized using the highest value at the cut off temperature to provide 100% fluorescence. The difference curves were derived by subtracting the melting profile of the control from the test sample melting profiles.

**Table 2:** Oligonucleotide primers used in HRMA qPCR.

Primer	Description	Sequence (5’-3’)^a^	Primer Pair: Product size
SK144	CCR5, S	CGAGCAAGCTCAGTTTACACC	
SK145	CCR5, AS	GCCATGTGCACAACTCTGACT	SK144 and SK145: 107 bp
SK210	F8-S1, S	ATGTCCTGTAGGGTCTGATCG	
SK211	F8-S1, AS	ACAACCATCCTAACCCGATG	SK210 and SK211: 87 bp
SK228	F8-S2, S	ACTGCCAATTCTTTTCATAGGTC	
SK229	F8-S2, AS	AGGCAGAGGGAAGTTTTGTC	SK228 and SK229: 150 bp
SK230	F8-S3, S	TCATGTCTGGTTCTCTTGTGTG	
SK231	F8-S3, AS	CTGCCTAAATGCACCAGAAC	SK230 and SK 231: 134 bp

^a^The oligonucleotide sequences for CCR5 were derived from GenBank accession number NG_012637 and for F8 from GenBank accession number NG_011403.

S: Sense, AS: Antisense.

Conditions for amplification of target sites F8-S1, -S2 and -S3 in the intron 1 locus were similar to above except for using a denaturation temperature for 95°C for 2 min for initial step, followed by 39 cycles consisting of a denaturation step at 95°C for 10s, and an annealing and extension temperature of 60°C for 30s. This was followed by a cycle consisting of 95°C for 30s and 60°C for 1’. The melting analysis was carried out between 65°C and 95°C with 0.2°C increments and 10s/step as described above.

### Generating mixes of qPCR products for dose-response curves

Following amplification, the two PCR products to be used for the standard curves were diluted in Bio-Rad 1x Precision Melt Buffer to give similar relative fluorescent units (RFUs) on the CFX96 thermocycler prior to mixing them in the intended proportions.

### TaqMan assay

Separate TaqMan probes, labeled with the fluorescent dye FAM and quenchers Zen and Iowa (IDT, USA), to detect modifications at F8-S1 and -S2 target sites were designed to bind across the spacer sequence between the left and right gRNA binding sites and used with the same primers as for HRMA (Table 3). The optimal annealing and extension step for TaqMan qPCR was determined by a preliminary temperature gradient experiment to identify the temperature that could provide the greatest discrimination between wildtype and mutant target sequence. The qPCR was carried out using the following cycling parameters: one step of 95°C for 3 min (hot start), followed by 40 cycles with a denaturation step of 95°C for 10s and an annealing cum extension step of 66°C for 30s. Serial dilutions of template gDNA from unmodified cells were used to generate standard curves. The same samples were also used for a TaqMan qPCR using primers SK237 and SK238 with FAM/Zen/Iowa-labeled probe SK239 for quantitation of the reference β-actin gene (Table 3).

**Table 3:** Oligonucleotide primers and probes used in TaqMan qPCR.

Primer/Probe	Description	Sequence (5’-3’)	Primer pair: Product size
SK210	F8-S1, S	ATGTCCTGTAGGGTCTGATCG	
SK211	F8-S1, AS	ACAACCATCCTAACCCGATG	
Heterologous/S1 Probe	FAM/Zen/Iowa-S1 probe Tm 62.6	ATGTAAGTCTGCTTGGAGGAAGGT	SK210 and SK211: 87 bp
SK228	F8-S2, S	ACTGCCAATTCTTTTCATAGGTC	
SK229	F8-S2, AS	AGGCAGAGGGAAGTTTTGTC	
Homologous/S2 Probe	FAM/Zen/Iowa-S2 probe3 Tm 62.2	TGCTCCTCCAGAATCTAGAGACTG	SK228 and SK229: 150 bp
SK237	β-actin, S	AGAAAATCTGGCACCACACC	
SK238	β-actin, AS	TGATCTGGGTCATCTTCTCG	SK237 and SK238: 120 bp
SK239 Probe	β-actin FAM/Zen/Iowa-probe Tm 63.5	TTCTACAATGAGCTGCGTGTGG	Probe for use with SK237 and SK238

S: Sense, AS: Antisense.

Results of qPCR were analyzed using Bio-Rad CFX Manager (version 3.0) software. The Bio-Rad software uses the Pfaffl method to determine relative amounts of target molecules between different test samples. This requires estimating the PCR efficiencies for amplifying the target region of interest with the efficiency for amplifying the β-actin reference gene. To obtain the efficiencies, we used serial dilutions of a known positive sample. From regression analysis of these dose-response curves to obtain the slopes, the software estimates the efficiencies (E) of the qPCR reactions. The relative quantities (ΔΔCq) are determined by the formula: E_target_^ΔCq_target^/ E_reference_^ΔCq_reference^. These values were then normalized to user designated samples (e.g., to zero or unedited control, or to the highest expressor) for graphical representation. Additional details of calculations and determination of standard deviations for the normalized quantities are available in the Help menu of the software.

### Homologous recombination (HR)-specific PCR (HR-PCR)

The HR-specific primers were designed to amplify across the right and/or left homology arms of the donor plasmid by situating one of the primers in the genome and the other within the donor plasmid sequence across the right or left homology arm (Table 4). The optimal cycling parameters of HR-PCR were determined using preliminary temperature gradient experiments. The optimized HR-PCR for all primer pairs for CCR5-donor plasmid consisted of a denaturation and hot-start step of 98°C for 2 min, followed by 40 cycles consisting of 98°C for 10s and 67.6°C for 1’. The optimized HR-PCR for detecting HDR at the F8 target site consisted of a denaturation and hot-start step of 98°C for 3 min, followed by 40 cycles consisting of 98°C for 10s, 67.6°C for 10s, and 72°C for 40s. The PCR products were analyzed by agarose gel-electrophoresis for size confirmation.

**Table 4:** HR-PCR for CCR5 and TaqMan qPCR for F8 to detect site specific recombination of pDonor-CCR5 and pDonor-F8, respectively.

Primer/Probe	Description	Sequence (5’-3’)	Primer pair: Product size
SK106	β-actin, S	CATGTACGTTGCTATCCAGGC	
SK107	β-actin, AS	CTCCTTAATGTCACGCACGAT	SK106 and SK107: 250 bp
SK178	CCR5 locus, S	AGGGGTGAGGTGAGAGGATT	
SK179	pDonor-CCR5, AS	GGGAGTGAATTAGCCCTTCC	SK178 and SK179 span LHA: 1,628 bp
SK181	pDonor-CCR5, S	GGGCTGTCCCTGATATCAAAC	
SK182	CCR5 locus, AS	AAGGGTTTCTCCAATCTGC	SK181 and SK182 span RHA: 1,107 bp
SK218	F8 locus, S	ATGGAGGTCAGAAGCCATTG	
SK219	pDonor-F8, AS	AACAGCTCCTCGCCCTTG	SK218 and SK219 span LHA: 839 bp
SK240	FAM/Zen/Iowa-probe Tm 69.9	CCTGTGGCTGCTTCCCACTGATAAAA	Probe for use with SK218 and SK219

S: Sense, AS: Antisense.

The oligonucleotide sequences for were derived from GenBank accession numbers: NM_001101 (β-actin), NG_012637 (CCR5) and NG_011403 (F8).

### TaqMan HR-qPCR

We also designed a qPCR to detect HDR at the F8 S1, S2 or S3 target sites using the TaqMan probe SK240 together with oligonucleotide primers SK218 and SK219 (Table 4). The following PCR conditions were used: Initial hot-start at 98°C for 2’ followed by 40 cycles of 98°C for 10s and 66°C for 1 min.

The results were analyzed using Bio-Rad CFX Manager software as described above for TaqMan assay. Serial dilutions of a known HR positive sample were used to estimate the efficiency of PCR amplification of the target region of interest. The β-actin gene was used as the reference gene. The relative quantities (ΔΔCq) were estimated by the CFX Manager as described earlier for the TaqMan assay but referenced to the zero baseline.

### Next generation sequencing (NGS) and sequence analysis using Python

The genomic loci targeted by TALENs or RGENs were amplified by PCR using the same primer pairs as for HRMA (Table 2). PCR products were purified using MinElute PCR Purification Kit (Qiagen. Cat.No. 28004) as per the recommended protocol to recover amplicons as small as 70 bp in length. Barcoded sequencing libraries were prepared from the PCR products using the Ion Plus Fragment Library Kit (Life Technologies, ThermoFisher Scientific, USA) according to the supplier’s instructions, and sequenced using the Ion Torrent Proton sequencer (Life Technologies, ThermoFisher Scientific, USA) at the Genomics Core Facility of Saint Louis University. The sequence data were aligned to the human genome using the Torrent Mapping Program (TMAP) to generate BAM and indexed BAM files for visualization with the Integrated Genome Browser.

The Python programming language was used to design a module for NGS analysis (ngsAnalysis_v1.0.py in S1 Appendix). This module (given the sequence of target, sense and antisense primers, and a stringency factor) contains functions that extract sequences from the input NGS data file corresponding to a particular target locus based on the presence of sense and antisense primer sequences. The sequence between the primers is then compared to that of the input wild type target sequence using the dynamic programming algorithm for edit-distance, which provides the minimum number of changes (substitutions, insertions or deletions) required to convert one string to another. The module identifies insertions and deletions based on comparison of size of the query sequence to that of wild type, and a minimal value (stringency factor) of edit-distance (to compensate for ‘background’ mutations resulting from sequencing artifacts), and outputs text files with a summary of number of insertions, deletions, and percentage of all mutations, as well as separate files of deletions and insertion sequences and their sizes.

### Statistical analysis

Two molecular clones were tested for each RGEN or TALEN construct. The transfections were done in duplicate for each molecular clone. Real time PCR also used duplicates. Thus a minimal of four replicates were used for deriving averages and standard deviations for each data point. The Student’s t-tests were used for statistical analyses to compute significance of difference between two sample means while ANOVA tests were used for comparing more than two sample means.

## Results

### HRMA reveals modification at the CCR5 target site by TALENs

**Fig 1:**
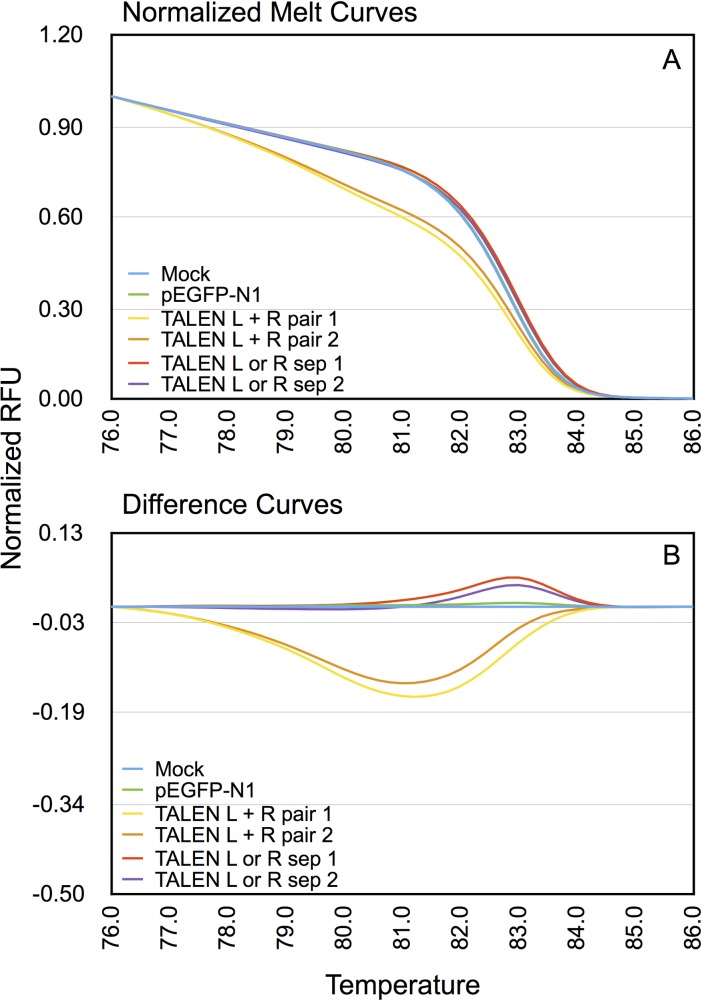
Detection of genome editing at the CCR5 locus by TALENs using HRMA. The gDNA of HEK293T cells mock-transfected, or transfected with pEGFP-N1 alone or with the left (L) or right (R) TALEN or both (L + R). The gDNA of transfected cells were subjected to qPCR using primers SK144 and SK145 and analyzed by HRMA post-amplification as described in Material and Methods. A) Normalized melt curves from indicated transfections. The y-axis shows normalized relative fluorescence units (RFU) while the x-axis depicts temperature (with a 0.2°C resolution between data points as detailed in Materials and Methods). Only 1°C temperature increments are shown for clarity. B) Difference curves were obtained by subtracting the normalized melting profile of each test sample from that of mock-transfected control. Two molecular clones were tested for each of the right and left TALENs. The pairs of TALENs used were designated as pair 1 or pair 2. sep: Separate transfections with either left or right TALEN alone.

HEK293T cells were mock-transfected or transfected with pEGFP-N1 (Clontech, USA) (that encodes EGFP) alone, or with the left CCR5-targeting TALEN expression construct, or the right TALEN expression construct, or both. The target region in CCR5 was amplified from gDNA isolated from transfected cells and analyzed by HRMA as described in Materials and Methods. The results are shown in Fig 1A. The HRMA of amplified gDNA of mock-transfected cells, or cells transfected with pEGFP-N1 alone or with either left or right TALEN, showed a normalized melting profile indistinguishable from each other. In contrast, HRMA of amplified gDNA of cells transfected with both left and right TALEN encoding constructs showed a distinct melting profile with a lower Tm. The difference curves, where the melting profile of control mock-transfected amplified gDNA was subtracted from left and right TALEN-transfected cells, are shown in Fig 1B. These results demonstrate a distinguishable melting profile for those amplified gDNA obtained from cells transfected with both right and left TALENs. The control amplification of β-actin region from the same gDNA samples did not exhibit any unique melting profile among the different test groups.

To compare HRMA to traditional methods used to identify efficacy of genome editing reagents, we also carried out a Surveyor Nuclease Assay (SNA) using a commercial kit (Transgenomics, USA) as described in Materials and Methods. This kit also contains a control in which two PCR products differ in their sequences by one nucleotide (contain either G or C). Mixing the two products, followed by denaturation and reannealing results in a single-stranded region at the site of mismatch that is amenable to cleavage by the Surveyor endonuclease resulting in digestion products with sizes of 416 bp and 217 bp (S6B Fig). In contrast to the GC mismatch control, PCR across CCR5 target site would be expected to result in a heterogeneous product due to different types of mutations expected as a result of NHEJ repair. Consistent with this, digestion of the PCR product across the putative target site in cells receiving both left and right TALENs resulted in a faint smear instead of a distinct band (yellow rectangle, S6A Fig). No corresponding smear was observable in the samples derived from mock-transfected cells or cells transfected with individual TALENs or just pEGFP-N1.

### HRMA can be used to distinguish the efficiency of RGENs targeting F8 intron 1

**Fig 2:**
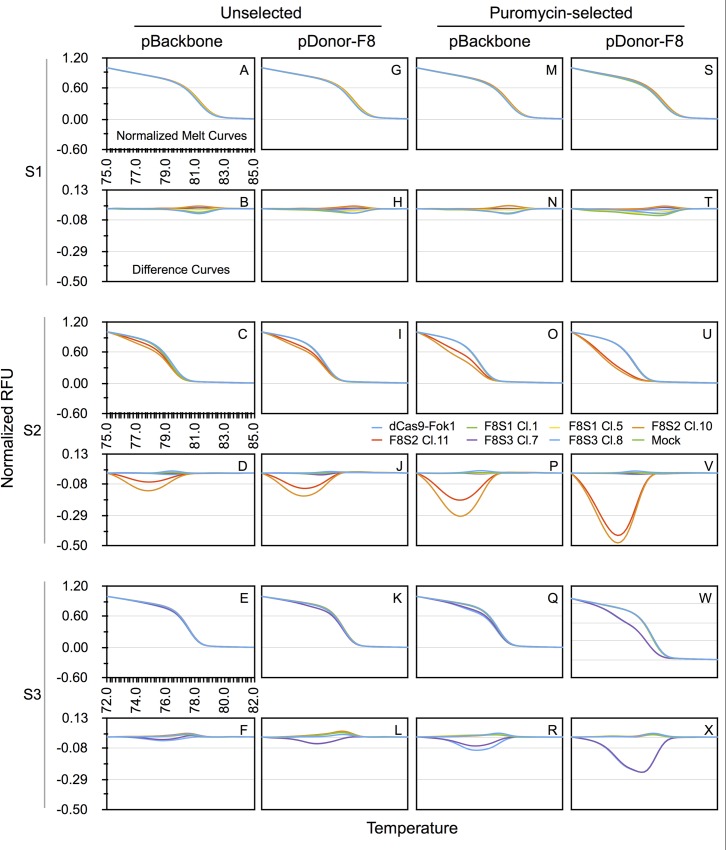
HRMA reveals differing efficiencies of RGENs targeting F8 intron 1 and its sensitivity for detection can be dramatically enhanced by cotransfection with a donor template followed by drug selection for the transfected population. HEK293T cells were transfected with pSQT1313_F8S1 (F8S1), pSQT1313_F8S2 (F8S2) or pSQT1313_F8S3 (F8S3) together with pSQT1601 (which encodes dCas9-FokI) and either pBackbone or pDonor-F8 as shown. The gDNAs were isolated from either unselected or puromycin-selected samples as indicated. The gDNAs were subjected to qPCR using primers specific for F8-S1, -S2 or -S3 target sites and analyzed by HRMA post-amplification as described in Material and Methods. Normalized melt curves and the corresponding difference curves (alternating rows) are shown for each identified test condition (unselected vs puromycin-selected, pBackbone vs pDonor-F8) and F8 target (depicted as S1, S2 or S3). The y-axis shows normalized RFU while the x-axis depicts temperature. The temperature range used for analysis are shown only for the top left melting curve plot for each target. The same range was used for F8-S1 and -S2 target sites while a slightly lower range was used for F8-S3 target site PCR product. To facilitate comparison between plots, the y-axis range was kept the same for all plots of a given kind (melting profiles vs difference curves). The gRNA from each transfection was evaluated with all pairs of primers (F8-S1, -S2 or -S3 specific primer pairs, Table 2). The graph legend shown in the middle identifies the construct used for transfection by line color. Thus each normalized melt curve plot shows results of HRMA analysis of eight pairs of transfections. In the difference curve plots, mock subtracted from itself are not shown. The same graph legend is therefore applicable for all the plots. Two molecular clones were tested for each plasmid construct. The molecular clones are referred to as Cl.x where x is the clone designation number. Error bars have been omitted for clarity but graphs with error bars are available in the DataFile.xlsx in S1 Appendix.

We also carried out HRMA analyses of amplified gDNA of HEK293T cells transfected with pSQT1601(that expresses CRISPR/dCas9-FokI), along with gRNA encoding constructs (pSQT1313_F8S1, pSQT1313_F8S2, or pSQT1313_F8S3) targeting one of three sites, F8-S1, S2, or S3, situated in intron 1 of human F8 locus (Table 1). The results shown in Fig 2A, 2C and 2E, indicate that the gDNA obtained from cells receiving pSQT1313_F8S2 site exhibited a distinct and discernible melting profile, while that obtained from pSQT1313_F8S3 treated cells, showed only a small deviation of the melting profile from gDNA of control unmodified target cells. The melting profile of amplified gDNA across S1 target site from cells receiving pSQT1313_F8S1 was not distinguishable from that of the controls. The difference curves derived from normalized melt curves for target sites F8-S1, -S2 and -S3 (Fig 2B, 2D and 2F) also lead to the same conclusions. The efficiencies of genome editing can be characterized as pSQT1313_F8S2 > pSQT1313_F8S3 > pSQT1313_F8S1.

### Detection of genome editing by HRMA is facilitated by selecting for transfected population

To decrease the background HRMA signal contributed by gDNA of untransfected cells in the pool, we selected the cells after transfection using puromycin. This was possible due to cotransfection of gRNA expressing plasmids with the pBackbone construct that encoded an RFP-Puromycin fusion gene. The results of normalized melting profiles of gDNA of puromycin-selected cells transfected with pSQT1313_F8S2 or pSQT1313_F8S3 exhibited greater differences from the control melting curves than those observed with unselected cells (Fig 2O and 2Q vs Fig 2C and 2E, respectively). In contrast, HRMA of pSQT1313_F8S1 transfected cells showed melting profile differences that were indistinguishable from control curves even after selection (Fig 2A vs Fig 2M). Comparison of difference curves (Fig 2N, 2P and 2R vs Fig 2B, 2D and 2F) also yielded the same conclusions as those derived from normalized melt curve profiles for F8-S2 and -S3 target sites. The relative Tm differences seen in unselected cells was maintained with puromycin-selected cells: i.e., pSQT1313_F8S2 > pSQT1313_F8S3 > pSQT1313_F8S1.

### Detection of genome editing by HRMA is enhanced by inclusion of donor plasmid with RGENs during transfection of HEK293T cells

DSB at the target site effected by the genome editing reagent should be amenable to HDR in the presence of an appropriate donor plasmid containing sequences with homology to genomic sequence on either side of the DSB. We therefore included pDonor-F8 (HR-F8LHA_GpA-RHA, S5B Fig) that contained left and right homology arms for homologous recombination in a parallel set of transfections of HEK293T cells (to pBackbone transfected cells described above) with pSQT1313_F8S1, pSQT1313_F8S2 or pSQT1313_F8S3 together with pSQT1601 (that expresses dCas9-FokI). We hypothesized that presence of pDonor-F8 would skew the repair pathway towards homology-mediated DNA repair instead of non-homologous end joining repair (NHEJ) and thereby decrease detection of NHEJ by HRMA. In contrast to our hypothesis, melting profiles of gDNA from unselected cells that received the pSQT1313_F8S2 or pSQT1313_F8S3 and pDonor-F8 (Fig 2I and 2K) demonstrated greater decrease in Tm than corresponding gDNA of cells receiving pBackbone construct (Fig 2C and 2E). Maximal decrease in Tm was seen with melting profiles of gDNA of cells receiving pDonor-F8 and selected using puromycin (Fig 2U and 2W). The difference curves of the corresponding melting profiles exhibit the same characteristics (Fig 2V vs 2P and Fig 2X vs 2R). Again, pSQT1313_F8S2 transfected cells provided the greatest difference in Tm, followed by pSQT1313_F8S3 transfected cells. The gDNA from pSQT1313_F8S1 transfected cells showed almost no evidence of target site modification as deduced from melting profiles or difference curves even for samples receiving pDonor-F8 and subsequent drug selection (Fig 2S vs 2M and Fig 2T vs 2N).

### HRMA can be used for quantitation of proportion of mutant gDNA in cells transfected with RGENs

**Fig 3:**
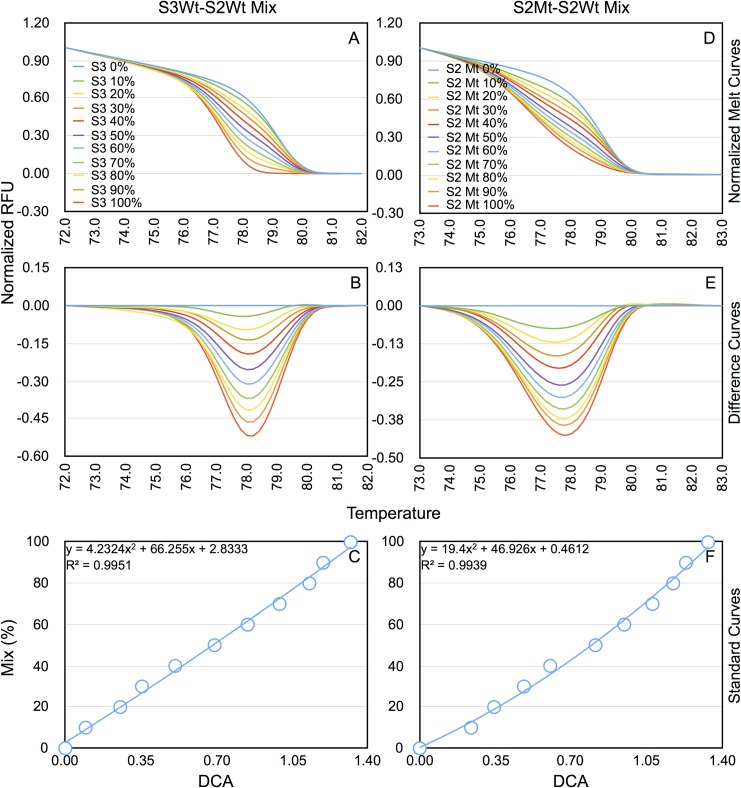
The difference curve areas show correlation with the percentage of Tm-shifted mutants in the population. Standard curves were generated using mixes of qPCR products from F8-S2 wild type (wt) and S3 wt sites (S3Wt-S2Wt mix), or F8-S2 Wt and F8-S2 mutant (Mt) sites in the proportions shown. (A) Normalized melt curves of mixes of S3 and S2 wild type PCR products (S3Wt-S2Wt mix). (B) Difference curves of the various mixes shown in panel A. (C) Standard curve derived by plotting (polynomial plot) areas enclosed by the difference curves (DCA) in panel B vs percentage of S3 wt PCR product in S2 wt PCR product. (D) Normalized melt curves of mixes of qPCR products of F8-S2 wt and F8-S2 mutant site (S2Mt-S2Wt mix) in the proportions indicated. (E) Difference curves of the various mixes shown in panel D. (F) Standard curve derived by plotting areas DCAs in panel E versus percentage of S2 mutant PCR product in S2 wt PCR product. The correlation coefficient and equations are shown for each standard curves in panels C and F. Please see text for additional details.

The results of the previous set of experiments showing differences in Tm between test and control samples for the same RGEN under different conditions (after selection for transfected cells or by cotransfection with a donor template for HDR) suggested that HRMA might be useful as a quantitative tool. To measure the proportion of mutants, it was necessary to establish a dose-response curve with mixtures of mutant target sequences with wild type target sequences. To mimic mixtures of mutant and wild type sequences, we first created mixes of separately amplified qPCR products from control gDNA using F8-S2 and S3 target specific primers (Table 2) to provide a range from 0% to 100% of S3 qPCR product in the background of S2 qPCR product in increments of 10%. The S3 product was used as a surrogate for the mutant S2 product as their Tms were comparable (~77°C). The S3 wild type (S3Wt)-S2 wild type (S2Wt) mixes were then analyzed by HRMA. The normalized melt curves and difference curves are shown in Fig 3A and 3B respectively. With increasing proportion of S3 qPCR product in the mix, the normalized melt curves exhibited an increasing difference in Tm from the control containing 100% S2 PCR product. We plotted the areas under the difference curves (DCA) (Fig 3C) against percentage of S3 in S3Wt-S2Wt mixes. Curve fitting revealed that a second order polynomial equation provided a correlation coefficient of 0.9951. Re-estimation of proportions from the equations gave values within 2 ± 1% of expected percentages (Table 5).

**Table 5:** Cross-application of standard curves to estimate proportion of mutant molecules with HRMA.

Nominal % in mix of PCR products	S3Wt-S2Wt STD Curve on self		S2Mt-S2Wt STD Curve on self		S3Wt-S2wt STD Curve on S2Mt-S2Wt DCA	S2Mt-S2Wt STD Curve on S3Wt-S2Wt DCA
	DCA^a^	%	DCA	%	%	%
100	1.32	97.7	1.33	97.6	98.7	96.2
90	1.19	87.9	1.23	87.7	90.8	84.0
80	1.13	83.1	1.17	82.1	86.3	78.2
70	0.99	72.7	1.08	73.5	79.1	66.0
60	0.84	61.8	0.95	62.2	69.3	53.9
50	0.69	50.6	0.81	51.5	59.5	42.1
40	0.51	37.6	0.60	35.8	44.4	29.3
30	0.35	26.9	0.48	27.6	35.7	19.5
20	0.25	19.9	0.34	18.9	26.1	13.6
10	0.09	9.1	0.24	12.7	18.8	5.0
0	0.00	2.8	0.00	0.5	2.8	0.5

^a^DCA: Difference Curve Area.

**Fig 4:**
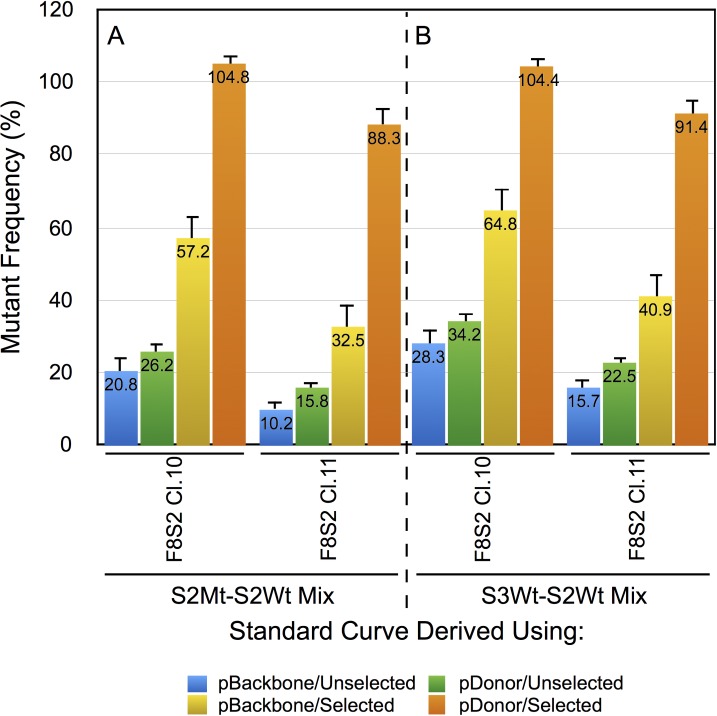
Standard curves using mixes containing either mutant molecules or a surrogate PCR product substituting for the mutant detect comparable levels of genome editing in RGEN transfected cells. HEK293T cells transfected with pSQT1313_F8S2 clone 10 or 11 together with pSQT1601, and either pBackbone or pDonor-F8. The percentage of mutants was determined from the DCAs of test samples (Fig 2) using standard curve equations shown in Fig 3C. (A) Estimation of proportion of mutants using S3Wt-S2Wt standard curve. (B) Estimation of proportion of mutants using S2Mt-S2Wt standard curve (Fig 3F). Error bars represent 1 SD.

The DCAs for pSQT1313_F8S2 clones 10 and 11 were determined for unselected or puromycin-selected samples that received either pBackbone or pDonor-F8. The proportions were then estimated using the polynomial equation referenced above. The results are shown in Fig 4B. Consistent with the normalized melting curve and the difference curve profiles, for all the different conditions evaluated, F8S2 clone 10 appeared to be more efficient than clone 11. The cells that received RGEN and pDonor-F8 showed higher percentages of mutants than those from samples that received pBackbone. The highest efficiency of genome editing (104.8%) was observed in cells that received pSQT1313_F8S2 clone 10 with pDonor-F8 plasmid and then selected with puromycin. In these samples the PCR product consisted of almost entirely mutated S2 target molecules (Fig 4B, brown bar for clone 10). pSQT1313_F8S2 clone 11 showed slightly lower amounts of mutant molecules (91.4%) under the same conditions.

**Fig 5:**
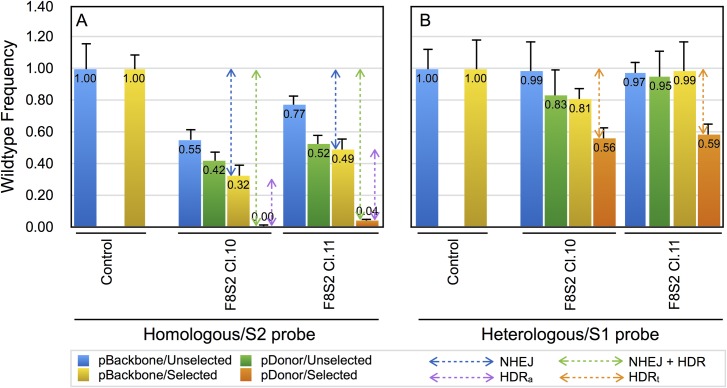
TaqMan assay to measure remnant quantity of wild type target sequence following genome editing with RGENs. The gDNA of HEK293T cells transfected with pSQT1313_F8S2 clone 10 or 11 together with pSQT1601, and either pBackbone or pDonor-F8 were assayed using TaqMan qPCR (as described in Materials and Methods). (A) TaqMan assay using F8-S2 specific probe (homologous/S2 probe). (B) TaqMan assay using F8-S1 specific probe (heterologous/S1 probe). The qPCR signal for unmodified cells, either unselected or puromycin selected were designated as controls and assigned a value of 1.0. The magnitude of reduction for samples receiving pBackbone was attributed to NHEJ repair (blue bidirectional arrows) while those from samples that received pDonor-F8 was attributed to a combination of both NHEJ and HDR DNA repair pathways (green bidirectional arrows). The apparent contribution of HDR (HDR_a_) repair pathway to the reduction in qPCR signal is shown by the bidirectional purple arrows. The contribution of true HDR (HDR_t_) repair pathway to the reduction in qPCR signal was using the heterologous/S1 specific probe and is shown by the bidirectional brown arrows in panel B. Error bars represent 1 SD.

Having identified a gDNA sample consisting of 100% mutant molecules from cells receiving pSQT1313_F8S2 clone 10, pDonor-F8 a second dose-response experiment was performed using mixtures of the S2 mutant qPCR (S2Mt) product with S2 wild type (S2Wt) qPCR product as for S3Wt-S2Wt mixes. Subsequent experiments using TaqMan assays (see below) confirmed that no wildtype sequences were detectable in the S2Mt product (Fig 5A). The normalized melting curves and the difference curves of these new mixes are shown in Fig 3D and 3E. Standard curves generated using DCAs of the mixes vs S2Mt percentage provided a correlation coefficient of 0.9939. Recalculation of input amounts in the S2Mt-S2Wt mixes from curve fitting polynomial equation again gave values within 2.3 ± 1% of expected percentages (Table 5).

We next cross-applied the equation obtained with S3Wt-S2Wt mix standard curve to the DCAs of S2Mt-S2Wt mixes and *vice versa*. The results are shown in Table 5. The S3Wt-S2Wt standard curve equation yielded within 5.8 ± 3.2% of expected percentages with DCAs of S2Mt-S2Wt (Table 5). Likewise, when S2Mt-S2Wt standard curve equation was used to estimate percentages of S3Wt-S2Wt mixes, it provided values that were within 5.7 ± 3.2% of expected percentages.

Using the equations generated by both sets of standard curves, we estimated the percentages of mutations in gDNAs of HEK293T cells receiving pSQT1313_F8S2 clone 10 or 11 under the different experimental conditions (unselected vs puromycin-selected, in the presence of pBackbone or pDonor-F8) (Fig 4). The proportion of mutants in F8-S2 target site estimated from DCAs using S3Wt-S2Wt standard curve was 28.3% for pSQT1313_F8S2 clone 10 and 15.7% for pSQT1313_F8S2 clone 11 for gDNA of transfected cells that received pBackbone and were unselected. With the S2Mt-S2Wt mix generated standard curve, the proportion of mutants estimated from DCAs was 20.8% for pSQT1313_F8S2 clone 10 and 10.2% for pSQT1313_F8S2 clone 11 for the same samples. For all experimental conditions tested (unselected vs puromycin-selected, pBackbone vs pDonor), pSQT1313_F8S2 clone 10 showed higher percentages (up to 9%) than those for clone 11 with the exception of puromycin-selected HEK293T cells that also received pDonor-F8 that showed about 104% of mutants for pSQT1313_F8S2 clone 10 with both standard curves. These results of estimation of proportion of mutants with the two different standard curves were consistent with the results of cross-application experiment of the standard curves shown in Fig 3. Thus, pSQT1313_F8S2 clone 10 appears to be more efficient than pSQT1313_F8S2 clone 11 for genome editing at the F8 locus. The most likely explanation for the difference between the two clones is the point mutation found within the middle oligo sequence of clone 11 (S1 Table, ‘C’ nucleotide shown in red).

A multi-factor ANOVA test was done to determine the statistical significance of differences in mutation frequencies between 1) the dimeric gRNA F8S2 clones 10 and 11, 2) the various conditions (pBackbone vs pDonor-F8 and selected vs unselected samples in different combinations), 3) the two standard curves, and 4) the possible interactions between the factors. The results of these analyses (ANOVAFig4.pdf in S1 Appendix) revealed that the mutation frequency means 1) between the two dimeric gRNA clones, 2) between selected and unselected samples, 3) between pBackbone and pDonor-F8 samples, and 4) between the two standard curves, were all statistically significant (p < 0.05). These results demonstrate that HRMA can be used to differentiate RGENs with different efficiencies of modification of target sites.

### TaqMan assay for independent assessment of genome editing

The above experiments with HRMA suggested sequence altered target sites based on differences in Tm between gDNA of control cells and those from RGEN or TALEN-transfected HEK293T cells. An alternative method to HRMA that measures proportion of mutant molecules, is to use a TaqMan qPCR assay to quantitate the wild-type sequences in gDNA of cells that were genome edited using TALENs or RGENs and then determine the proportion of mutant sites from the reduction in the amount of wild type sequence in the target PCR product.

We designed two probes for assessment of genome editing using the TaqMan assay. A homologous probe that binds over the F8-S2 target site (homologous/S2 probe) and a heterologous probe that binds to the F8 S1 neighboring site (heterologous/S1 probe) to estimate the proportion of wildtype in the different samples. The proportion of wild type sequence in gDNA of cells transfected with genome editing reagents is predicted to decrease for the homologous/S2 probe, but not for the heterologous/S1 probe, reflecting the efficiency of target site modification.

The gDNA was isolated from cells transfected with pSQT1313_F8S2 (clones 10 or 11) and pSQT1601 together with either pDonor-F8 or pBackbone. The gDNA isolated from both unselected and puromycin-selected cells were tested using the TaqMan assay using homologous/S2 probe and the heterologous/S1 probe with the indicated primer pairs (Table 3). The samples were also analyzed by a TaqMan qPCR for β-actin. The results of the TaqMan assay with homologous/S2 probe, normalized to β-actin (ΔΔCq) are shown in Fig 5A. The proportion of wild type sequence in the gDNA of unselected cells that received pBackbone was 55% and 77% for pSQT1313_F8S2 clones 10 and 11, respectively (Fig 5A, blue columns). For selected cells, the proportion of wild type was decreased further to 32% and 49% of the control, respectively for the same clones (Fig 5A, yellow columns).

We next determined the proportion of wild type S2 sites in cells that received pSQT1313_F8S2 (clone 10 or 11) and pDonor-F8 as template for recombination at the target site. Recombination at the F8-S2 target site by cotransfection of cells with pDonor-F8 also results in a decrease in the measured quantity of wild type target sequence as the donor plasmid that provides the template for HDR lacks the binding site for the probe. The results indicate that in unselected cells (Fig 5A, green columns) the wild type amount decreased to 42% and 52%, respectively for the two clones. For puromycin-selected cells, the proportion of wild type was dramatically decreased to near zero for both clones (Fig 5A, brown columns). It is to be noted that the DSBs that are modified by HDR with donor template lack the primer and probe binding sites and are therefore eliminated from the assay.

For all conditions tested, pSQT1313_F8S2 clone 10 treated samples exhibited a greater reduction in wildtype sequence than clone 11 indicating a greater efficiency of target site modification by clone 10. These differences between the clones were found to be statistically significant (ANOVA and paired T-test, p < 0.05). As for HRMA, ANOVA test for the TaqMan assay revealed that the differences in the means, between pBackbone vs pDonor transfected samples, and between selected vs unselected samples, were also statistically significant (p< 0.05, ANOVAFig5.pdf in S1 Appendix).

### Comparison of HRMA and TaqMan assay results

**Fig 6:**
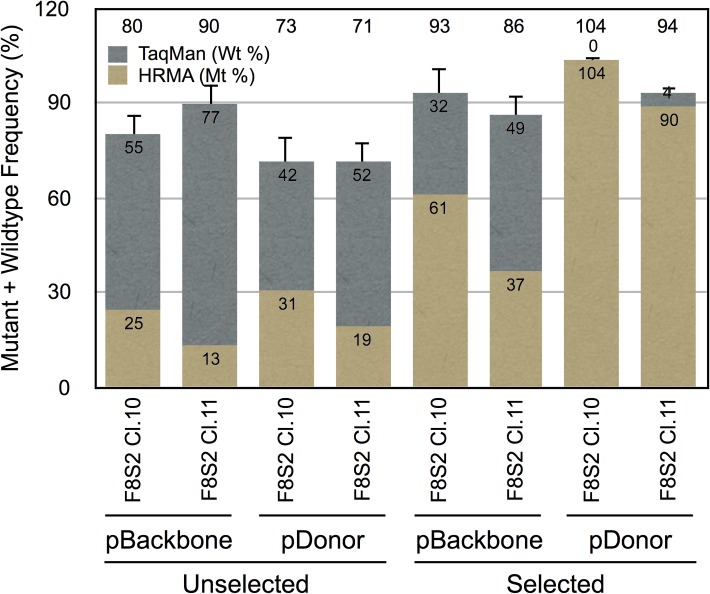
Stacked column graph showing sum of mutant and wild type frequencies at genome edited F8-S2 target sites in HEK293T cells. Mutant percentage (Mt %) within the population was measured using HRMA, and the remnant wild type percentage (Wt %) was measured by a TaqMan assay. This figure combines the results of the experiments described in Fig 4 (average of Fig 4A and 4B for the indicated categories) and Fig 5A. Two molecular clones, pSQT1313_F8S2 clones 10 (F8S2 Cl.10) or 11 (F8S2 Cl.11) were tested with pSQT1601, and either pBackbone or pDonor-F8 (pDonor) as shown. The cells were either unselected or selected using puromycin as indicated. The sum of the measured percentages is shown above the tops of the stacked columns.

The percentage of mutant target molecules determined from HRMA corresponds to those that have undergone NHEJ mediated DNA repair and exhibit a distinct Tm from unmodified controls. The TaqMan assay, in contrast to HRMA, measures the frequency of wild type sequences for target amplified with the same pair of primers as for HRMA. The reduction in wild type target sequence detected by the TaqMan assay can occur either due to NHEJ or due to HDR-mediated DNA repair involving exogenously added donor temple that lacks PCR amplifiable target sequence. Since HRMA and TaqMan assays provide complementary information (frequency of mutant vs that of wildtype), and the sum of the mutant and wildtype percentages for the same sample must equal 100%, the combined data are shown in a stacked column graph (Fig 6). We were able to account for 75% to 100% of the observed efficiencies of modification of target sites by RGENs under different experimental conditions (unselected vs selected and pBackbone vs pDonor-F8) from the sum of mutant and wildtype frequencies (Fig 6, numbers shown above the columns).

### Measurement of ‘true’ HDR by TaqMan qPCR

To directly measure the true contribution of HDR repair pathway for the decrease in TaqMan signal at the F8 S2 target site (HDR_t_), we designed a probe (heterologous/S1 probe) that bound to the spacer region between left and right gRNA binding sites for the F8-S1 target site. The S1 probe is 179 nt upstream of the spacer region of the F8-S2 target site. Indel mutations at the F8-S2 site are not expected to affect the binding of probe at the F8-S1 site. The pDonor-F8 design allowed its use for HDR at all three F8 target sites. Thus, HDR at the S2 site with template provided by pDonor-F8 will eliminate the binding of S1 probe as well as the S2 probe. To directly measure the contribution of HDR at F8-S2 site, or ‘true’ HDR, the same samples used for TaqMan qPCR using homologous/S2 probe were also subjected to TaqMan qPCR using the heterologous/S1 probe using the primer pair shown in Table 4. The results of this qPCR assay are shown in Fig 5B. The cells that received pBackbone together with F8S2 RGEN appeared to show a small decrease, but this decrease was not statistically significant (p = 0.107). As anticipated, the only statistically significant decrease in TaqMan signal for the S1 probe was observed for gDNA of puromycin-selected cells that received pDonor-F8 (p = 0.008 for clone 10 and p = 0.006 for clone 11). The S1 TaqMan signal was reduced from corresponding controls for pSQT1313_F8S2 clones 10 and 11 (Fig 5B) to 56% and 59%, respectively.

### Estimating NHEJ and HDR repair frequency

The reduction in wild type target sequence in samples that received pBackbone and RGEN (pSQT1313_F8S2 clone 10 or 11) can be attributed to NHEJ mediated DNA repair alone (Fig 5 blue bi-directional arrows). The reduction in wild type target sequence in samples that received pDonor and pSQT1313_F8S2 (clone 10 or 11) and detected using the homologous S2 probe, can be attributed to a combination of NHEJ and HDR mediated DNA repair pathways (HN). The apparent contribution of HDR_a_,was the difference between TaqMan quantitation between samples that received pDonor-F8 and the corresponding sample that received pBackbone (Fig 5 purple bi-directional arrows). The binding of the heterologous S1 Probe is not affected by NHEJ repair at S2 site but is only reduced by homologous recombination at S2 site in the presence of pDonor-F8. We refer to the measured reduction of binding of the heterologous S1 Probe as true HDR (HDR_t_) (Fig 5B, brown bidirectional arrows).

Table 6 summarizes the results of determinations of proportion of NHEJ, HDR_a_, HDR_t_, and combined NHEJ and HDR in HEK293T cells receiving pSQT1313_F8S2 clone 10 or 11 and either pBackbone or pDonor-F8. The equations for calculating NHEJ, apparent, and true HDR values are elaborated at the bottom of the table. The apparent HDR (HDR_a_) was 0.32 and 0.45 for pSQT1313_F8S2 clones 10 and 11, respectively while the true HDR (HDR_t_) was 0.44 and 0.41 for the same clones. The ratio of HDR_t_ to HN was determined to be 44 and 42.7%, respectively for the two clones. These results indicate that approximately 43% of DSBs introduced by RGENs are available for HDR-mediated DNA repair in HEK293T cells.

**Table 6:** Estimation of frequency of NHEJ, and apparent and true HDR following genome editing.

		Homologous / S2 Probe			Heterologous / S1 Probe		
		pBackbone	pDonor-F8		pBackbone	pDonor-F8	
Repair Pathways (Frequency)		N	HN	HDR_a_		HDR_t_	% HDR_t_ in HN
**RGEN**	**pSQT1313-F8S2 Cl.10**	0.68	1.00	0.32	na	0.44	44.00
	**pSQT1313-F8S2 Cl.11**	0.51	0.96	0.45	na	0.41	42.71

N: NHEJ; HN: NHEJ and HDR combined; na: not applicable.

N = (Ec—Eb)/Ec.

HN = (Ec-Ed_hom_)/Ec.

HDR_a_ = (HN—N)/Ec.

HDR_t_ = (Ec—Ed_het_)/Ec.

Where

Ec = TaqMan qPCR (ΔΔCq) measurement of unedited control sample

Eb = TaqMan qPCR (ΔΔCq) measurement of cells edited using pSQT1313-F8S2 and pBackbone.

Ed_hom_ = TaqMan qPCR using homologous/S2 Probe of cells edited using pSQT1313-F8S2 and pDonor-F8.

Ed_het_ = TaqMan qPCR using heterologous/S1 Probe of cells edited using pSQT1313-F8S2 and pDonor-F8.

% HDR_t_ = (HDR_t_/HN) x 100.

### HRMA shows correlation with NGS analysis of target sites

We next determined the sequence of the target sites modified by TALENs or RGENs by NGS. The gDNA of unselected cells transfected with the genome editing reagents and pBackbone were used to amplify the target sites using the primer pairs shown in Table 2 and the PCR products were sequenced using NGS technology as described in Materials and Methods. The sequence alignment results were analyzed using Integrated Genome Browser which confirmed that the deletion mutations were localized to the target site in gDNA of transfections that received RGENs or TALENs but not in gDNA of control transfections.

**Fig 7:**
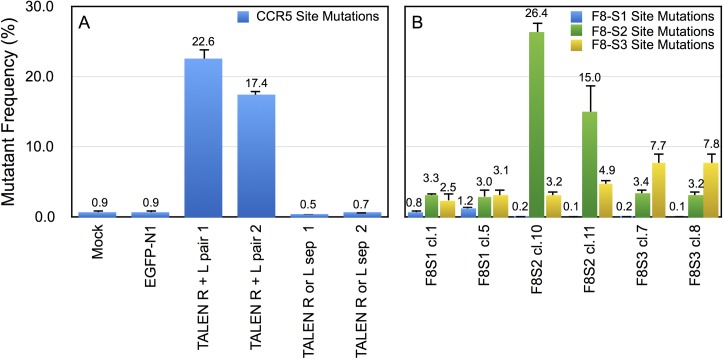
Determination of mutation percentage of target sites modified by TALENs targeting CCR5 or RGENs targeting F8-S1, S2 and S3 sites by NGS. HEK293T cells were transfected with plasmid constructs encoding TALENs or RGENs as described in Materials and Methods. The gDNA was isolated from transfected cells and amplified by PCR using the same primers used for HRMA analysis (Table 2) and subjected to NGS (Materials and Methods). The sequences were analyzed using Python scripts to obtain the percentage of mutations (base changes, insertions and deletions). Two plasmid expression clones were tested for each target site. L, R refer to left and right TALENs analyzed by NGS. (A) Mutation frequency at the CCR5 locus in cells mock-transfected cells or cells transfected with EGFP-N1 encoding plasmid alone or EGFP-N1 with either left TALEN, right TALEN (indicated as ‘sep’) or both. (B) Mutation frequency at F8 target sites S1, S2 or S3 in intron 1. The cells received pSQT1313_F8S1, pSQT1313_F8S2 or pSQT1313_F8S3, together with pSQT1601 and pBackbone. All gDNA used for NGS were obtained from unselected cells after transfection.

We used a Python module (Materials and Methods, ngsAnalysis_v1.0.py in S1 Appendix) to extract and enumerate wild type, deletion, and insertion mutations in each of the described target loci. The percentages of mutant sequences in the different target sites are shown in Fig 7. The NGS analysis of CCR5 target regions edited using TALENs revealed that in mock-transfected cells, or cells that received an EGFP encoding plasmid alone or with either the left or right TALEN, with an edit-distance cut off of 3, the background mutation detected was less than 1%. For cells that received both left and right TALENs, the percentage of mutations detected was 17 to 22-fold higher in two independent pairs of clones tested. This suggested that 17% to 22% of gDNA had indel mutations at the target site.

When we analyzed gDNA of cells transfected with pSQT1313_F8S1, pSQT1313_F8S2 and pSQT1313_F8S3 by NGS of target sites in intron 1, with the same edit-distance cut off, we obtained different background levels for each site. The controls for background mutation detection were sequences of the same target region from either untransfected HEK293T cells or cells transfected with the other RGENs than the one being analyzed.

Consistent with the HRMA and TaqMan results, the F8-S2 target site showed the greatest number of mutations (26.4% and 15.0% for pSQT1313_F8S2 clones10 and 11, respectively), followed by F8-S3 (7.7% and 7.8% for pSQT1313_F8S3 clones 7 and 8, respectively) and F8I-S1 (0.8% and 1.2% for pSQT1313_F8S1 clones 1 and 5, respectively). Accounting for approximately 3.2% background mutation for F8-S2 site in unmodified controls, or from cells modified by a different RGEN, the efficiencies for pSQT1313_F8S2 clones10 and 11 would be 23.2% and 11.8%, respectively. For pSQT1313_F8S3 clones 7 and 8, the average background subtracted values was 4.3% and 4.4% respectively. For pSQT1313_F8S1 clones 1 and 5, the background subtracted values were 0.65% and 1.05%, respectively.

The mutation percentages determined by NGS lie between the two estimates from HRMA experiments described above (Fig 4) using equations from two different standard curves. The CCR5 target site modified using TALENs showed a mutation percentage comparable to what was seen at the F8-S2 site (23.2% and 18.2% for the left and right TALEN clones pairs 1 and 2, respectively).

**Fig 8:**
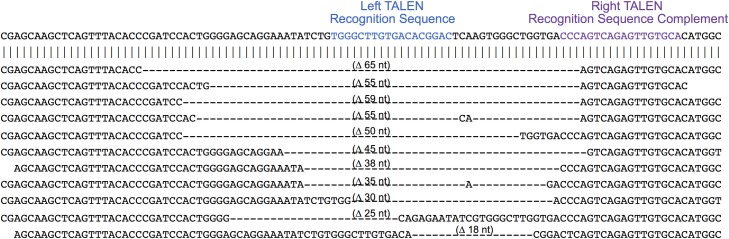
Deletion mutations at CCR5 locus identified by NGS. The wild type sequence is shown on the top. The left and right TALEN recognition sequences are shown in blue and purple, respectively. Deletions are indicated with dashed lines. The sizes of the deletions are shown above the dashed lines. A randomly chosen range of deletions of different sizes are shown.

**Fig 9:**
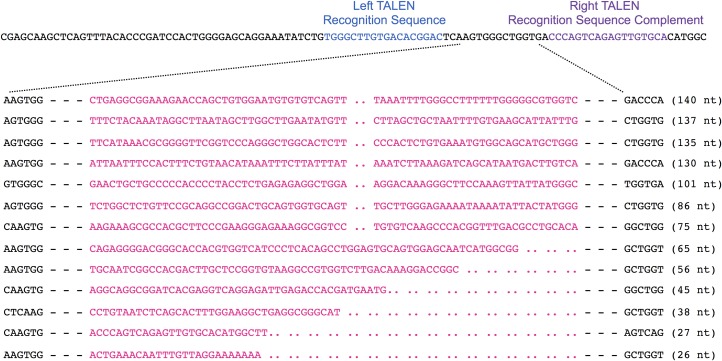
Insertion mutations at the CCR5 locus identified by NGS. The wild type reference target sequence is shown on the top. The left and right TALEN recognition sequences are shown. The inserts, in pink, are situated between the 6-mer sequences (in black) that map to the wild type sequence. Some insert sequences were shortened (represented by a pair of dots) to allow accommodation in the space available. The actual sizes of the inserts are shown to the right of the sequences in brackets.

**Fig 10:**
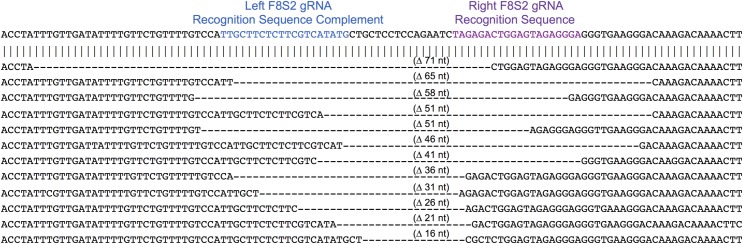
Deletion mutations in at the F8-S2 target locus identified by NGS and Python scripts. The left and right gRNA recognition sequences are shown in blue and purple, respectively. The left S2 gRNA recognition sequence is the complement of sequence highlighted in blue color. Deletions are indicated with dashed lines. The sizes of the deletions are shown above the dashed lines. A randomly chosen range of deletions of different sizes are shown.

**Fig 11:**
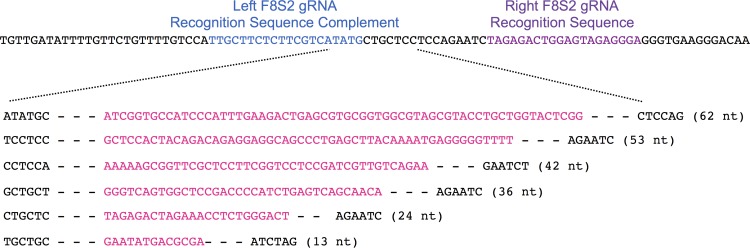
Insertion mutations identified at the F8-S2 target site identified by NGS. A sampling of insertions of different sizes are shown. The wild type reference target sequence is shown on the top. The left and right gRNA recognition sequences are shown. The left S2 gRNA recognition sequence is the complement of sequence highlighted in blue color. The inserts, in pink, are situated between the 6-mer sequences (in black) that map to the wild type sequence. The sizes of the inserts are shown to the right of the sequences in brackets.

The deletions and insertions extracted using Python Scripts were visually inspected to exclude false positives and then aligned with the wild type target sequence. A sampling of the range of deletion and insertion mutations observed at the CCR5 locus are shown Figs 8 and 9, respectively. The range of deletions and insertions observed at the F8-S2 loci are shown in Figs 10 and 11, respectively.

**Fig 12:**
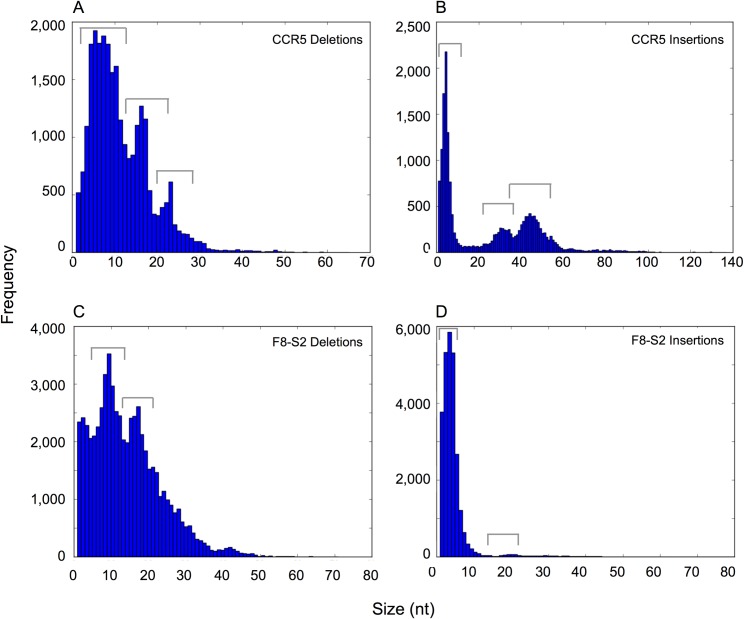
Frequency distribution of deletion and insertion mutations in gDNA of cells that received TALENs targeting the CCR5 target site or RGEN targeting F8-S2 site identified by NGS. The NGS files were analyzed using Python to separately sort deletion and insertion mutations. Multiple copies of the same mutation were eliminated to provide separate sets of unique deletions and insertions. The sizes of deletion and insertion mutations, obtained from the difference in size of the mutant with the wildtype sequence, were written into a text file and then plotted as described in the text. The frequency distribution of deletions and insertions at the CCR5 (A and B, respectively) or the F8-S2 (C and D, respectively) are shown. The most common groups of sizes are indicated by the ‘staples’ over the columns.

To identify the most common sizes of insertions and deletions, the frequency distribution of unique sequences with distinct sizes of deletions were extracted using the scripts in the Python module (ngsAnalysis_v1.0.py in S1 Appendix) at the CCR5 and F8-S2 target sites and histograms generated using ‘matplotlib’ 2D plotting library in Python. The results are shown in Fig 12. While large deletions (66 bp size deletion at the CCR5 target site and 79 bp deletion at the F8-S2 site) were detectable at both target sites, majority of deletions clustered around 6–12 bp or 14–18 bp. Although infrequent, some large insertions (Figs 9 and 11) could be discerned at the F8-S2 and CCR5 target sites. Insertion mutations constituted less than 4% of all mutations.

### Confirmation of homologous recombination of donor template at DSB sites

**Fig 13:**
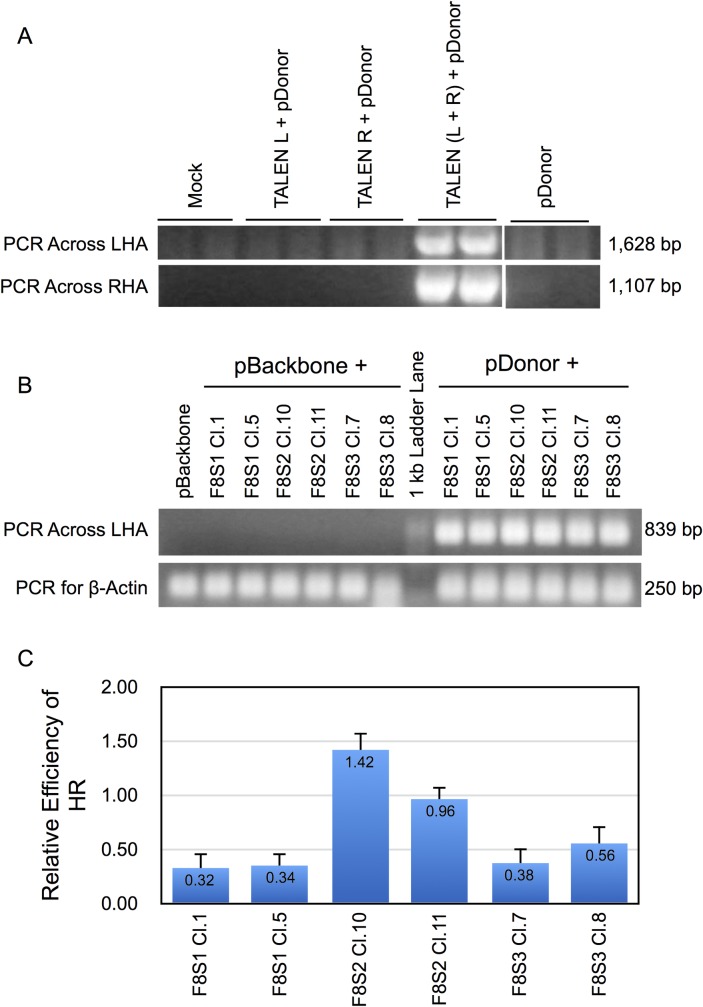
Detection of site-specific recombination in TALEN or RGEN transfected HEK293T cells by HR-PCR and HR-qPCR. **(A)** HEK293T cells were transfected with TALENS targeting CCR5 locus and pDonor-CCR5. The gDNA isolated from the puromycin-selected cells were subjected to PCR designed to amplify the sequence across the right (RHA) or left homology arms (LHA) into the genome using primer pairs shown in Table 4 and resolved using agarose gel electrophoresis. (B) HEK293T cells were transfected with pSQT1313_F8S1, pSQT1313_S2 or pSQT1313_S3 targeting F8-S1, -S2 or -S3 sites, respectively together with pSQT1601 and either pBackbone or pDonor-F8. The gDNA isolated from puromycin-selected cells were subjected to PCR across LHA as described in Materials and methods and resolved using agarose gel electrophoresis. (C) The gDNA isolated from puromycin-selected cells shown in ‘B' were subjected to TaqMan qPCR designed to amply the sequence across the LHA using primer pairs and probe shown in Table 4. The efficiency of HR relative to zero is depicted on the y-axis. Error bars indicate 1 SD.

Finally, to confirm HDR-mediated homologous recombination at the intended target sites in the presence of a donor template DNA, gDNA isolated from puromycin-selected cells transfected with RGENs or TALENs, and either pBackbone, or a donor plasmid to a provide template for HDR, were subjected to HR-PCR using primers designed to amplify across the left and/or right homology arms present in the donor plasmid into the neighboring genome sequence (Materials and Methods). Agarose gel electrophoretic analysis (Fig 13A) of the qPCR products showed bands of the appropriate size only for samples that received the donor template and both left and right TALENs indicating the specificity of HDR. Similarly, HR-PCR of gDNA of RGEN transfected cells showed appropriately sized bands for samples that received donor template while no corresponding product was seen for samples that received pBackbone (Fig 13B).

Since HR-PCR is not quantitative, (note the similar intensity of PCR products on agarose gel electrophoresis in Fig 13B), for all three RGEN transfected cells), we also carried out a TaqMan HR-qPCR to estimate the efficiency of site-specific recombination at the three different F8 target sites. Fig 13C shows results of HR-qPCR across LHA at F8-S1, S2 and S3 loci for gDNA of cells transfected with pSQT1313_F8S1, pSQT1313_F8S2 or pSQT1313_F8S3, pSQT1601 and pDonor-F8. Parallel transfections with pBackbone instead of pDonor-F8 served as controls. The samples were assayed by TaqMan qPCR for β-actin. The relative quantities of site-specific PCR product with respect to β-actin are shown in Fig 13C. The results showed that the samples that received pSQT1313_F8S2 (together with pDonor-F8) showed the highest amounts (1.42 ± 0.19 for clone 10 and 0.96 ± 0.08 for clone 11). These amounts were statistically significant from each other and the HR-qPCR results obtained for F8-S1 and -S3 target sites. Only background levels of TaqMan signal were detectable for samples that received pBackbone instead of pDonor. HDR at F8-S3 site (0.38 and 0.56 for pSQT1313-F8S3 clones 7 and 8, respectively) showed a tendency to be higher than that of F8-S1 site (0.32 and 0.34 for pSQT1313-F8S1 clones 1 and 5 respectively). Thus the results of HR-qPCR mimicked those of HRMA and supported the conclusion that the efficiencies of genome editing could be characterized as pSQT1313_F8S2 > pSQT1313_F8S3 > pSQT1313_F8S1. The lower sensitivity of HR-qPCR to HRMA can be attributed in part to the larger size of the PCR product (839 bp).

## Discussion

### Qualitative assessment of efficacy of genome editing reagents using HRMA

HRMA has been proposed as an alternative to gel-electrophoresis or DNA sequencing to screen for genetic variants (including pathologic variants, single nucleotide polymorphism typing etc) [23,25]. For e.g., it has been used for detection of mutations in KRAS, BRAF and PIK3CA in metastatic colorectal cancer [26–29]. HRMA has been recently adapted for the detection of mutations at target sites introduced by genome editing reagents in *Aedes aegypti* and zebrafish [30]. The Parant laboratory has extensively evaluated the utility of HRMA to distinguish homozygous and heterozygous mutants from wild type alleles in zebrafish. In one study they were able to distinguish point mutations, small deletions and retroviral insertions from wild type sequence using HRMA [25]. In a more recent study [21], they were able to detect chimerism as low as 5% in G0 animals using this assay. They demonstrated that HRMA could readily detect two distinct alleles in the background of wild type sequence. A mix of three alleles and wild type resulted in less distinct melting profiles but again were easily distinguishable from the wild type. Dahlem and coworkers injected one cell zebrafish embryos with mRNA encoding TALENs targeted to distinct exons and were able to detect modified exons using an appropriate primer pair across the target with HRMA [22]. They identified a variety of indels at the different target sites consistent with NHEJ repair.

The above referenced investigators used different approaches to extract information from melting profiles. The Parant laboratory used derivative transformations of normalized melting profiles [21,25]. This resulted in distinct Tm-shifted peaks. Deletion alleles shifted the peaks to the left. They were able to readily distinguish up to two alleles in this fashion. Larger than two alleles in the mix with wild type resulted in less distinct peaks with lower resolving power. Dahlem and coworkers in contrast used normalized melting curves without further transformation and determined possible number of alleles by the number of ‘humps’ in the melting curve profile [22]. Other investigators used difference curves, as in our study, to identify mutant alleles in a target.

Our study extends the studies of Parant and coworkers, and Dahlia et al., in that we used HRMA after transient transfection of HEK293T cells in which a multitude of ‘alleles’ are expected to be present. Despite this heterogeneity, we were able to readily distinguish the comparative efficiencies of different genome editing agents in normalized melting profiles as well as in the difference curves.

We initially tested the efficacy of HRMA to detect mutations introduced by TALENs in the CCR5 locus just 3’ of the coding exon. A distinct melting profile was discernible only in HRMA of gDNA obtained from transfections that received both left and right TALENs. We compared these results with that of Surveyor Nuclease assay (SNA). Although results of SNA were in concurrence with that of HRMA, the results of HRMA were unambiguous compared to the ‘smear’ obtained in test samples with the SNA. The advantage of HRMA over SNA is that the analysis is carried out as part of the PCR run (closed single tube approach), while SNA requires that the sample be first melted, reannealed and then digested with single-strand specific endonuclease prior to resolution by polyacrylamide or agarose gel electrophoresis. Moreover, visualization of bands after gel electrophoresis requires one to generate sufficiently large amounts of the PCR product for the endonuclease digestion step.

We next extended the HRMA studies to RGENs targeting intron 1 of F8 gene and were able to determine that RGENs targeting F8-S2 site exhibited the highest efficiency of target site modification compared to those targeting the other two sites (Fig 2). We were able to further increase the sensitivity of HRMA by reducing the contribution of gDNA from untransfected cells by including a plasmid encoding a selection marker (pBackbone) and selecting for transfected population prior to HRMA analysis. When we included the pDonor-F8 plasmid to provide an appropriate template for repair at DSB site, the proportion of mutant molecules in the amplified PCR product was further increased. The above results demonstrate that HRMA is an effective qualitative tool for quickly identifying efficient genome editing reagents.

### Quantitative assessment of efficacy of genome editing reagents

The results described above alluded to the possibility that HRMA could be used to quantitate the proportion of mutant molecules in a PCR product. We hypothesized that difference curves areas would vary in relation to the proportion of mutant species. We tested this hypothesis by producing mixtures of wild type target PCR product with PCR product which exhibited a similar Tm as mutant PCR product (S3Wt-S2Wt mix) to generate standard curves. The standard curve using S3Wt-S2Wt mixes initially allowed us to identify the 100% mutant S2 sample. We were then able to use this S2MT sample to generate a more ‘authentic’ mix (S2Mt-S2Wt mix) and to compare both types of mixes in standard curves to quantitate the frequency of mutants in other test samples. The two types of mixes used to generate standard curves may exhibit different characteristics due to the possibility of formation of heteroduplexes in the S2Mt-S2Wt mix. This could in turn skew the melting profiles of S2Mt-S2Wt when compared to that of S3Wt-S2Wt mix. Surprisingly, however: 1) We could cross apply the S3Wt-S2Wt and S2Mt-S2Wt standard curves (Table 5) i.e., one standard curve could substitute for the other. 2) The values obtained from the cross application were generally within 6% of the expected percentages. 3) Both standard curves identified comparable levels of mutant species in the test samples and showed similar trends (Fig 4A vs 4B). However, the small but consistent differences in mutation frequency values in test samples (Fig 4A vs 4B) seen with the two different standard curves were determined to be statistically significant (multi-factor ANOVA, p < 0.05). We attribute this difference between the standard curves to inherent difficulties in generating mixes with precise ratios of mutant (or a surrogate) and wildtype target molecules based on RFUs of final PCR products. For a given nominal amount of mutant in the mix, the DCA was slightly higher with S2Mt-S2Wt when compared to the corresponding mix with the S3Wt-S2Wt mix. From this, one can surmise that for a given DCA the S2Mt-S2Wt standard curve based estimation would tend to be lower than that estimated with the S3Wt-S2Wt standard curve. A solution to this issue would be to use absolute quantification using droplet digital PCR (ddPCR) for generation of more accurate mixes for standard curve generation.

Despite the potential caveats of using a heterologous PCR product, the surrogate S3Wt PCR product was able to effectively substitute for the S2Mt product for quantitation in the test samples. In the absence of a 100% mutant sample with which to generate a dose-response curve, we suggest using a surrogate PCR product (with a Tm that is ~2°C lower than the wild type product) for quantitative HRMA that might be able to provide a reasonable first approximation of mutation frequency in the test samples.

### Alternative methods to HRMA for measuring efficiency of target site modification

The T7 endonuclease I (T7EI) assay is a commonly used method for detection of target site modification by genome editing reagents. The T7EI assay is similar to the Surveyor nuclease assay that we used in this study. Lin et al., [31]used clever modifications of the T7EI assay to determine relative contributions of NHEJ and HDR for repair of DSBs and demonstrated that synchronizing cells in the cell division cycle enhanced HDR in cultured cell lines and primary cells. Zhu et al., [32] also used the T7EI assay to identify target site modification in NHEJ knock-down silk worms. The T7EI assay was used as a qualitative tool by Zhu et al., while Lin et al., used the assay for quantitation of NHEJ and HDR at genome edited target sites.

A novel PCR based approach was described by Yu et al., [33]to distinguish wild type and mutant alleles at genome edited target sites. Two pairs of primers were used, one pair contained a primer located over the putative DSB site. Modification at the target site selectively abrogated amplification with the inner pair of primers. The procedure required cloning of the PCR products into T-vector for detection of genome edited target sites.

T7EI and the T-vector cloning assays, therefore, require additional steps of manipulation after PCR of target loci. HRMA in contrast is a single tube/well method in which a controlled melting step is appended post amplification of target region of interest followed by *in silico* analysis. HRMA, therefore, offers some obvious advantages over the above referenced alternative methods.

### Exogenous donor template reveals high efficiency of DSB induction by genome editing reagents

The relative amounts of mutants and wild type sequences in the qPCR product from a given target site affects HRMA. Not all DSBs introduced by the genome-editing reagents result in mutations at the cut site. DSBs, in the absence of a donor template, are believed to undergo NHEJ mediated DNA repair [11,13]. HRMA reveals those DSBs that are repaired through the NHEJ DNA repair pathway. Some of the DSBs, depending on the stage of the cell cycle (S/G2), can conceivably be restored to a wild type sequence if a sister chromatid can provide the template for HDR. The restored sequences at DSBs would be scored as wild type by HRMA and the results would therefore underestimate the true efficiency of cutting by the genome editing reagents. When an exogenous template (that lacks the target sequence recognized by genome editing reagents, and the binding sites for the primers used in HRMA) replaces the target site as a result of successful HDR, those ‘silent’ DSBs can be unmasked. Thus, if all the ‘silent’ DSBs are eliminated from HRMA by HDR with an exogenous template, only the mutant sites remain with no contribution DSBs to the wild type by restoration, and HRMA would reveal all remnant amplifiable mutant target molecules. In other words, successful recombination with the donor template results in elimination of the wild type target for amplification by PCR. This increases the relative amount of mutant population in the PCR product that would manifest as an enhanced HRMA signal. For puromycin-selected cells that received donor template along with the genome editing reagent (Fig 4, brown columns) HRMA indicated that 90%-100% (depending on the particular F8-S2 targeting RGEN) of the remaining amplifiable target molecules consisted of mutant sequences.

### TaqMan qPCR assay to measure contribution of NHEJ and HDR in repair pathways of DSBs

To confirm the above explanation of the HRMA results, we designed a TaqMan qPCR assay for quantitation of wild type sequences across the F8-S2 target site. The results showed, as anticipated, that only 0% - 4% of wild type target molecules could be detected by TaqMan qPCR, in puromycin-selected cells transfected with F8-S2 targeting RGEN together with a donor plasmid (Fig 5A, brown columns). By using a second TaqMan probe that bound to an adjacent site, FV8-S1, outside of the primary target cut site (FV8-S2), referred to as heterologous/S1 probe here, we were able to directly determine the contribution of HDR to DNA repair in the presence of donor template sequence (Fig 5B brown bi-directional arrow and columns). The combination of HRMA and TaqMan assay results clearly demonstrated that a large fraction of DSBs (~42–43%) are available for HDR (Table 6) but are masked by restoration to wild type.

### Comparison of HRMA and TaqMan assays for measurement of efficiency of genome editing reagents

Since HRMA detects the mutant population in PCR amplified genome edited target region, and TaqMan (with the homologous probe) measures the frequency of wildtype population in the same PCR product, the sum of mutants and wild type frequencies is expected to approach 100%. Surprisingly, for unselected samples, which would also include the wild type sequences of untransfected population in addition to those from transfected population, we were able to account for only 80 to 90% of expected contributions of mutant and wildtype populations. For F8-S2 targeting RGEN (clone 10), HRMA estimated about 25% mutant frequency while TaqMan measured 45% mutant species (Fig 6, column 1, subtracting wildtype frequency from control). One possible explanation could be that the wildtype sequences of untransfected cells in the population may have had a negative impact on HRMA detection leading to an underestimation of mutant population frequency. This premise could not be supported by the NGS data which aligned closely with HRMA results. We are therefore unable to account for the ‘missing’ 10–20% in the unselected cells that received pBackbone. In contrast to the unselected samples, for selected samples, the discrepancy from the expected 100% of the sum of mutant and wildtype frequencies was less pronounced and could account for over 93% for cells transfected with pBackbone.

The additional decrease in wildtype detected by TaqMan in cells transfected with RGEN and pDonor-F8 can be explained by the replacement of the target site with that of the donor template. Since the donor template lacks sites for primers used for amplification of F8-S1 or S2 regions, the TaqMan assays records this as a decreased signal in comparison to the unmodified target in the control.

Another possible explanation for differences between HRMA and TaqMan assays is the disparate effect of insertions and deletions on the two assays. Both types of mutations can abrogate the binding of the TaqMan probe. In contrast, deletions reduce the Tm of the PCR product while insertions tend to increase the Tm. Furthermore, point mutations and indels that may not change the Tm could nevertheless affect probe binding. Thus HRMA inherently tends to under estimates mutation frequency.

Despite these potential drawbacks, HRMA has some potential advantages over the TaqMan assay for detecting target site modification. It appears to be more robust than TaqMan when using the same primer pair for target region amplification. TaqMan assays require a second amplification for a reference gene for normalization. The variations in the measurement of target and reference genes in TaqMan are subject to ‘error’ propagation leading to reduced ability to detect modest decreases in target quantities (see wildtype frequency estimation of unselected samples using heterologous /S1 probe, Fig 5).

Miyaoka et al have addressed some of the inherent limitations of the TaqMan qPCR assay by using droplet digital PCR (ddPCR) [34]. They used ddPCR in conjunction with distinct probes to measure NHEJ or HDR events in a variety of cultured cell lines and induced pluripotent stem cells. They also measured NHEJ and HDR frequency in different target loci within the same cell type. They demonstrated that the choice of HDR or NHEJ repair pathways depends on not only on the type of cell but also on the particular target locus within a given cell type. While HRMA and/or TaqMan can be done with realtime thermocyclers, ddPCR requires access to a droplet generator and a reader together with associated reagents that add considerably to the cost of performing such assays. In our study, we used HRMA or a TaqMan assay with homologous probe to measure NHEJ events and a TaqMan assay with a heterologous probe to measure ‘true’ HDR events. Further studies are necessary to determine the advantages and disadvantages of the different methods used to measure NHEJ and HDR frequencies following genome editing in different cell types or at different target loci within a given cell type.

### Differing efficiencies of RGENs targeting F8 intron 1

Parant and co-investigators found that higher GC content within guide sequences of RGENs was usually predictive of greater probability of successful modification at the target site [21]. In our studies we used dimeric gRNAs to recruit the dCas9-Fok1 endonuclease for DSB at a desired target locus. The average GC content of both guide RNAs for the three F8 sites S1 (52.5%), S2 (45%), or S3 (37%) did not show correlation with target modification efficacy of the RGENs. Since the length of the spacer sequences between the binding sites of the left and right gRNAs were shown to influence efficacy [9], we also examined this among the different F8 RGENs. The RGENs targeting F8-S1 and S3 had a spacer distance of 15 bases while that targeting F8-S2 had 16 bases separating the recognition sites of the left and right gRNAs. Thus, neither the GC content or the spacer distance could explain the dramatic difference in efficacy of the three F8 RGENs. We next scanned the target sequence encompassing all three sites and found four potential cytosine methylation sites (CpG dinucleotide). Both left and right gRNA recognition sequences of pSQT-F8S1 targeting F8 S1 had one CpG dinucleotide while only one CpG dinucleotide was found in one of the gRNAs of each of other two RGENs targeting F8-S2 and -S3. Additional studies are required to determine if any of these sites are in fact methylated *in vivo* and if the methylation pattern can account for functional differences between the RGENs.

### NGS analysis of genome edited target loci

We independently confirmed the reliability of HRMA and TaqMan assays to determine efficacy of genome editing reagents by NGS of target sites. The maximum size of deletions detected using TMAP alignment to the human genome sequence was about 25 nt. We suspected that we were missing larger deletions and insertions at the target cut sites. We explored alignment algorithms (Bowtie2, BWA, BWA-MEM) on the Galaxy online resource (usegalaxy.org) in conjunction with varying many of the input parameters to allow detection of larger deletions and insertions. As these trials proved ineffective in identification of larger deletions or insertions, we designed and tested an in-house Python module capable of sorting mutant from wild type sequences at a given target locus and estimating the proportion of insertions, deletions and total mutations. The percentage of mutations at the target locus determined from NGS data using the Python module was concordant with the estimation by HRMA and TaqMan analysis. For a given stringency, the background mutation percentages detected by the Python module varied with different target loci. Thus, for a stringency using edit distance of 3, the F8-S2 and S3 target sequences exhibited higher background levels of ‘mutation’ than F8-S1 target sequence (Fig 7). We attribute this variation to the sequence ambiguity generated by the NGS technology with different target sequences (e.g., not all bases may be identified in runs of particular nucleotides). Further refinements in the Python module may be able to address these issues.

To summarize, in this study, we investigated the utility of HRMA for measuring mutation frequency at genome edited target loci in a mixed population of cells after transient transfection. To the best of our knowledge, this is the first demonstration of a quantitative HRMA assay for measurement of mutation frequency. We corroborated the mutation frequency estimation by HRMA with NGS of the target sites. We showed, using HRMA, that RGENs targeting F8, even though situated close to each other, showed dramatic differences in their efficiency in inducing DSBs (F8-S1 targeting RGEN was much less effective than the F8-S2 RGEN). This implies that one should evaluate several RGENs to identify the most effective one. We describe a novel modification to the TaqMan assay that uses homologous and heterologous probes to respectively measure combination of NHEJ and HDR frequency or pure HDR frequency at genome edited target sites. Collectively, our data indicates that genome editing reagents can engender DSBs at high efficiency in HEK293T cells but a significant proportion of these are likely masked by reversion to wild type as a result of HDR with sister chromatids. Inclusion of a donor plasmid to provide a template for HDR (that simultaneously eliminates PCR quantifiable target) reveals these cryptic DSBs, by serving as a surrogate for the sister chromatid, and facilitates determination of the true efficacy of genome editing reagents.

## Supporting Information

S1 Appendix A zip file of additional files. DataFile.xlsx contains the data used for generation of figures and tables and also t-tests on the data. ANOVA analysis reports for Fig 4 and Fig 5 are in ANOVAFig4.pdf and ANOVAFig5.pdf. The NGS.Zip file contains next generation Fasta sequence files (NGSfastafiles.zip), the python module used for NGS analysis (ngsAnalysis_v1.0.py) and output files of NGS analysis (ngsAnalysisDataOutputFiles.zip). S1 Appendix also contains the gel image files (Gels.zip) showing gels with entire lanes of those shown cropped in Fig 13 and original and ‘inverted’ gels for S6 Fig.
(ZIP)

S1 Fig Output from ZiFit Targeter outlining the scheme for creation of left and right CCR5 TALENs. The top row of rectangles depicts the TALE repeat units and the repeat variable di-residue (RVD) with the associated nucleotide specificity. Adjacent repeats were ligated in four sequential steps as shown to create the left and right TALE sequences that were then cloned into the eukaryotic expression plasmid vectors JDS71 and JDS74, respectively. Additional details of cloning (e.g., restriction enzymes used for directional cloning and assembly of repeats) are available in the protocol accompanying the Addgene TALEN Assembly Kit.
(TIF)

S2 Fig Construction of dimeric gRNA encoding construct targeting intron 1 of human coagulation factor VIII (F8). (A) Cloning steps for creation of plasmid pSQT1313_F8S2. The dimeric gRNA expression construct was created by annealing oligos to generate left, middle and right oligo duplexes (AnnealedLTOD, AnnealedMTOD and AnnealedRTOD) specific for F8 S2 locus (S1 Table). These were then combined and ligated into BsmB1 digested pSQT1313 to generate pSQT1313_F8S2. An identical strategy was used for generation of dimeric gRNA expression constructs for targeting F8-S1 (pSQT1313_F8S1) and F8-S3 (pSQT1313_F8S3) sites. Please refer to Material and Methods for additional details. (B) Plasmid map of pSQT1313_F8S2. The right and left S2 gRNAs, the Csy4 recognition and cleavage sites, and other salient features are shown in the dimeric gRNA expression construct.
(TIF)

S3 Fig Cloning steps for creation of plasmid pDonor-CCR5 (HR-LHA-2-RHA-LGpA). The donor plasmid for homologous recombination at the CCR5 target site was generated as outlined. The left and right homology arms (LHA and RHA) were generated by PCR of genomic DNA using primers listed in S2 Table. These fragments were inserted using either In-Fusion (i.e., ligation-independent) cloning (LHA) or via the introduced restriction enzyme sites (RHA). A second internal expression cassette consisting of HIV-1 LTR promoter driving EGFP and bGH poly(A) was generated by SOE-PCR and inserted at the XbaI site by ligation-independent cloning. Please refer to Material and Methods for details.
(TIF)

S4 Fig Cloning steps for creation of plasmid pDonor-F8 (HR-F8LHA_GpA-RHA). The donor plasmid was generated using the steps depicted in this flow chart. Briefly, it consists of amplifying the right homology arm (RHA) from genomic DNA and inserting it into the multi-cloning site between BamHI and SphI restriction enzyme sites in pBackbone. The left homology arm containing the F8 first coding exon was amplified from human gDNA and fused in frame with EGFP coding sequence and terminated with bGH poly(A) signal sequence using splicing by overlap amplification. This fragment was introduced into the multiple cloning site for the left homology arm (LHA) in pBackbone. The oligonucleotide primers used for generating pDonor-F8 cloning are listed in S3 Table. Please refer to Material and Methods for additional details.
(TIF)

S5 Fig Plasmid maps of donor plasmids to effect HDR at genome edited sites. (A) pDonor-CCR5 (HR-LHA-2-RHA-LGpA). This plasmid contains the left (CCR5_LHA) and right (CCR5_RHA) homology arms to effect site specific recombination across the TALEN target cut site, an additional internal expression cassette consisting of the HIV-1 long terminal repeat (HIV-1 LTR) promoter driving enhanced green fluorescent protein (EGFP) and the bovine growth hormone (bGH) polyA site. (B) Plasmid map of pDonor-F8 (HR-F8LHA_GpA-RHA). This plasmid contains the left (F8_LHA) and right (F8_RHA) homology arms to effect site specific recombination across the F8 S1, S2 or S3 cut sites, EGFP in frame with F8 coding exon 1 and the bGH polyA sequence. Both donor plasmids contain an expression cassette consisting of the elongation factor-1 alpha (EF1-alpha) promoter driving a fusion gene composed of red fluorescent protein (RFP), self-cleaving picornavirus protease (T2A) and puromycin N-acetyl-transferase (PuroR) and terminated with the simian virus 40 (SV40) polyA signal. Other elements required for replication (ori) and selection (AmpR) in bacteria are also shown.
(TIF)

S6 Fig Surveyor Nuclease Assay for detection of genome-editing at CCR5 locus. A) HEK293T cells were transfected mock transfected or transfected with an EGFP encoding plasmid (EGFP) or left (L) TALEN, right (R) TALEN or both. Two pairs of left and right TALENs were tested. gDNA from each of the transfections were subjected to PCR using primer pair SK214 (5’-TGCTGTTCTATTTTCCAGCAA-3’) and SK215 (5’-CAGATGCCAAATAAATGGATGA-3’). These primers amplify a 247 bp product between nt 8728 and nt 8974 of CCR5 RefSeq (GenBank accession no. NG_012637). The PCR products were then digested with Surveyor Nuclease (Transgenomics Inc, USA) as per the recommended protocol and resolved on a 3% agarose gel. The region showing a smear of digestion products is enclosed within the yellow rectangle. B)‘G’ and ‘C’ controls from the Surveyor Nuclease kit produce a PCR product of size 632 bp. Upon mixing and reannealing, Surveyor nuclease digestion yields products of size 416 bp and 217 bp.
(TIFF)

S1 Table Oligonucleotide primers used for creation of dimeric-gRNA encoding plasmids targeting FVIII intron 1 sites S1, S2, and S3. (DOCX)

S2 Table Oligonucleotide primers used for creation of pDonor-CCR5. (DOCX)

S3 Table Oligonucleotide primers used for creating pDonor-F8. (DOCX)
