# Supplementary material for: Non-Homologous End Joining and Homology Directed DNA Repair Frequency of Double-Stranded Breaks Introduced by Genome Editing Reagents
Source: PLoS One. 2017 Jan 17;12(1):e0169931. doi: 10.1371/journal.pone.0169931 (PMC5241150; doi:10.1371/journal.pone.0169931)
Supplement: S1 Appendix — DataFile.xlsx contains the data used for generation of figures and tables and also t-tests on the data. ANOVA analysis reports for Fig 4 and Fig 5 are in ANOVAFig4.pdf and ANOVAFig5.pdf. The NGS.Zip file contains next generation Fasta sequence files (NGSfastafiles.zip), the python module used for NGS analysis (ngsAnalysis_v1.0.py) and output files of NGS analysis (ngsAnalysisDataOutputFiles.zip). S1 Appendix also contains the gel image files (Gels.zip) showing gels with entire lanes of those shown cropped in Fig 13 and original and ‘inverted’ gels for S6 Fig. (ZIP) [file pone.0169931.s001.zip › ANOVAFig4.pdf]

```
 * Encoding: UTF-8.

UNIANOVA mutation BY ruler source selection scissor
  /METHOD=SSTYPE(3)
  /INTERCEPT=INCLUDE
  /PLOT=PROFILE(source*selection selection*source source*scissor scissor*sourc
e selection*scissor scissor*selection)
  /EMMEANS=TABLES(OVERALL)
  /EMMEANS=TABLES(ruler)
  /EMMEANS=TABLES(source)
  /EMMEANS=TABLES(selection)
  /EMMEANS=TABLES(scissor)
  /EMMEANS=TABLES(ruler*source)
  /EMMEANS=TABLES(ruler*selection)
  /EMMEANS=TABLES(ruler*scissor)
  /EMMEANS=TABLES(source*selection)
  /EMMEANS=TABLES(source*scissor)
  /EMMEANS=TABLES(selection*scissor)
  /EMMEANS=TABLES(ruler*source*selection)
  /EMMEANS=TABLES(ruler*source*scissor)
  /EMMEANS=TABLES(ruler*selection*scissor)
  /EMMEANS=TABLES(source*selection*scissor)
  /EMMEANS=TABLES(ruler*source*selection*scissor)
  /PRINT=ETASQ DESCRIPTIVE
  /CRITERIA=ALPHA(.05)
  /DESIGN=ruler source selection scissor ruler*scissor ruler*selection ruler*s
ource
    scissor*selection scissor*source selection*source.
```

Univariate Analysis of Variance

Notes

Output Created		21-NOV-2016 12:09:09
Comments		
Input	Data	C:\Users\schuelkem\Desktop\2016-11-17 Kumar, Srinivas\Data (v0).sav
	Active Dataset	DataSet1
	Filter	<none>
	Weight	<none>
	Split File	<none>
	N of Rows in Working Data File	62
Missing Value Handling	Definition of Missing	User-defined missing values are treated as missing.
	Cases Used	Statistics are based on all cases with valid data for all variables in the model.

Notes

Syntax	UNIANOVA mutation BY ruler source selection scissor<br>/METHOD=SSTYPE(3)<br>/INTERCEPT=INCLUDE<br>/PLOT=PROFILE (source*selection selection*source source*scissor scissor*source selection*scissor scissor*selection)<br>/EMMEANS=TABLES (OVERALL)<br>/EMMEANS=TABLES (ruler)<br>/EMMEANS=TABLES (source)<br>/EMMEANS=TABLES (selection)<br>/EMMEANS=TABLES (scissor)<br>/EMMEANS=TABLES (ruler*source)<br>/EMMEANS=TABLES (ruler*selection)<br>/EMMEANS=TABLES (ruler*scissor)<br>/EMMEANS=TABLES (source*selection)<br>/EMMEANS=TABLES (source*scissor)<br>/EMMEANS=TABLES (selection*scissor)<br>/EMMEANS=TABLES (ruler*source*selection)<br>/EMMEANS=TABLES (ruler*source*scissor)<br>/EMMEANS=TABLES (ruler*selection*scissor)<br>/EMMEANS=TABLES (source*selection*scissor)<br>/EMMEANS=TABLES (ruler*source*selection*scissor)<br>/PRINT=ETASQ DESCRIPTIVE<br>/CRITERIA=ALPHA(.05)<br>/DESIGN=ruler source selection scissor ruler*scissor ruler*selection ruler*source<br>scissor*selection scissor*source selection*source.

Notes

Resources	Processor Time	00:00:03.09
	Elapsed Time	00:00:01.16

Between-Subjects Factors

		N
ruler	AUTH	31
	SURR	31
source	B	30
	D	32
selection	S	32
	Un	30
scissor	C10	30
	C11	32

Definitions

AUTH = S2Mt-S2Wt Standard Curve
SURR = S3Wt-S2Wt Standard Curve
B = pBackbone
D = pDonor-F8
S = Selected
Un = Unselected
C10 = pSQT1313-F8S2 clone 10
C11 = pSQT1313-F8S2 clone 11

'Ruler' refers to standard curve used for estimation of mutation frequency

'Source' refers to plasmid used in transfection

'Selection' refers to unselected or drug selected samples

'Scissor' refers to clones of RNA-guided endonuclease

Descriptive Statistics

Dependent Variable: mutation

ruler	source	selection	scissor	Mean	Std. Deviation	N
AUTH	B	S	C10	57.2500	6.21623	4
			C11	32.6125	6.02207	4
			Total	44.9313	14.33643	8
		Un	C10	20.8433	3.31675	3
			C11	10.1900	1.86689	4
			Total	14.7557	6.15112	7
		Total	C10	41.6471	20.04212	7
			C11	21.4013	12.67612	8
			Total	30.8493	19.02101	15
	D	S	C10	104.8200	2.27002	4
			C11	88.3450	4.36112	4
			Total	96.5825	9.37602	8
		Un	C10	26.2100	1.81305	4
			C11	15.8175	1.80123	4
			Total	21.0138	5.80151	8
		Total	C10	65.5150	42.06183	8
			C11	52.0813	38.89045	8
			Total	58.7981	39.74378	16

Descriptive Statistics

Dependent Variable: mutation

ruler	source	selection	scissor	Mean	Std. Deviation	N
	Total	S	C10	81.0350	25.79367	8
			C11	60.4788	30.18533	8
			Total	70.7569	29.12674	16
		Un	C10	23.9100	3.67959	7
			C11	13.0038	3.45433	8
			Total	18.0933	6.59453	15
		Total	C10	54.3767	34.76584	15
			C11	36.7413	32.12175	16
			Total	45.2745	34.06181	31
SURR	B	S	C10	64.7850	5.64206	4
			C11	40.9525	6.25546	4
			Total	52.8688	13.88147	8
		Un	C10	28.3067	3.72296	3
			C11	15.6975	2.32477	4
			Total	21.1014	7.26281	7
		Total	C10	49.1514	20.01818	7
			C11	28.3250	14.18871	8
			Total	38.0440	19.69934	15
	D	S	C10	104.3725	1.74462	4
			C11	91.3700	3.54266	4
			Total	97.8713	7.41536	8
		Un	C10	34.2650	1.96819	4
			C11	22.5250	2.11164	4
			Total	28.3950	6.55366	8
		Total	C10	69.3188	37.51357	8
			C11	56.9475	36.89812	8
			Total	63.1331	36.50878	16
	Total	S	C10	84.5788	21.51069	8
			C11	66.1613	27.35714	8
			Total	75.3700	25.60557	16
		Un	C10	31.7114	4.08661	7
			C11	19.1113	4.18877	8
			Total	24.9913	7.63331	15

Descriptive Statistics

Dependent Variable: mutation

ruler	source	selection	scissor	Mean	Std. Deviation	N
		Total	C10	59.9073	31.36612	15
			C11	42.6363	30.78584	16
			Total	50.9932	31.78025	31
Total	B	S	C10	61.0175	6.81360	8
			C11	36.7825	7.22396	8
			Total	48.9000	14.23520	16
		Un	C10	24.5750	5.16284	6
			C11	12.9438	3.53219	8
			Total	17.9286	7.25601	14
		Total	C10	45.3993	19.63432	14
			C11	24.8631	13.48030	16
			Total	34.4467	19.37498	30
	D	S	C10	104.5963	1.88947	8
			C11	89.8575	4.01801	8
			Total	97.2269	8.19319	16
		Un	C10	30.2375	4.64833	8
			C11	19.1713	4.01944	8
			Total	24.7044	7.09078	16
		Total	C10	67.4169	38.55138	16
			C11	54.5144	36.70812	16
			Total	60.9656	37.60457	32
	Total	S	C10	82.8069	23.01651	16
			C11	63.3200	27.98349	16
			Total	73.0634	27.07840	32
		Un	C10	27.8107	5.50843	14
			C11	16.0575	4.86863	16
			Total	21.5423	7.83766	30
		Total	C10	57.1420	32.65513	30
			C11	39.6888	31.09384	32
			Total	48.1339	32.79660	62

Tests of Between-Subjects Effects

Dependent Variable: mutation

Source	Type III Sum of Squares	df	Mean Square	F	Sig.	Partial Eta Squared
Corrected Model	64783.487[a]	10	6478.349	398.475	.000	.987
Intercept	138509.109	1	138509.109	8519.527	.000	.994
ruler	519.916	1	519.916	31.979	.000	.385
source	11254.153	1	11254.153	692.229	.000	.931
selection	40396.434	1	40396.434	2484.736	.000	.980
scissor	3699.553	1	3699.553	227.555	.000	.817
ruler * scissor	.136	1	.136	.008	.928	.000
ruler * selection	21.810	1	21.810	1.341	.252	.026
ruler * source	33.215	1	33.215	2.043	.159	.039
selection * scissor	243.668	1	243.668	14.988	.000	.227
source * scissor	104.370	1	104.370	6.420	.014	.112
source * selection	6966.908	1	6966.908	428.526	.000	.894
Error	829.150	51	16.258			
Total	209258.548	62				
Corrected Total	65612.637	61				

a. R Squared = .987 (Adjusted R Squared = .985)

Estimated Marginal Means

1. Grand Mean

Dependent Variable: mutation

Mean	Std. Error	95% Confidence Interval	
		Lower Bound	Upper Bound
47.442	.514	46.410	48.474

2. ruler

Dependent Variable: mutation

ruler	Mean	Std. Error	95% Confidence Interval	
			Lower Bound	Upper Bound
AUTH	44.542	.726	43.084	45.999
SURR	50.343	.726	48.885	51.801

3. source

Dependent Variable: mutation

source	Mean	Std. Error	95% Confidence Interval	
			Lower Bound	Upper Bound
B	33.919	.741	32.432	35.406
D	60.966	.713	59.535	62.397

4. selection

Dependent Variable: mutation

selection	Mean	Std. Error	95% Confidence Interval	
			Lower Bound	Upper Bound
S	73.063	.713	71.632	74.494
Un	21.821	.741	20.334	23.308

5. scissor

Dependent Variable: mutation

scissor	Mean	Std. Error	95% Confidence Interval	
			Lower Bound	Upper Bound
C10	55.196	.741	53.709	56.683
C11	39.689	.713	38.258	41.120

6. ruler * source

Dependent Variable: mutation

ruler	source	Mean	Std. Error	95% Confidence Interval	
				Lower Bound	Upper Bound
AUTH	B	30.285	1.045	28.186	32.384
	D	58.798	1.008	56.774	60.822
SURR	B	37.553	1.045	35.454	39.652
	D	63.133	1.008	61.109	65.157

7. ruler * selection

Dependent Variable: mutation

ruler	selection	Mean	Std. Error	95% Confidence Interval Lower Bound	95% Confidence Interval Upper Bound
AUTH	S	70.757	1.008	68.733	72.781
	Un	18.326	1.045	16.228	20.425
SURR	S	75.370	1.008	73.346	77.394
	Un	25.316	1.045	23.217	27.415

8. ruler * scissor

Dependent Variable: mutation

ruler	scissor	Mean	Std. Error	95% Confidence Interval Lower Bound	95% Confidence Interval Upper Bound
AUTH	C10	52.342	1.045	50.243	54.441
	C11	36.741	1.008	34.718	38.765
SURR	C10	58.050	1.045	55.951	60.149
	C11	42.636	1.008	40.613	44.660

9. source * selection

Dependent Variable: mutation

source	selection	Mean	Std. Error	95% Confidence Interval Lower Bound	95% Confidence Interval Upper Bound
B	S	48.900	1.008	46.876	50.924
	Un	18.938	1.086	16.758	21.118
D	S	97.227	1.008	95.203	99.251
	Un	24.704	1.008	22.681	26.728

10. source * scissor

Dependent Variable: mutation

source	scissor	Mean	Std. Error	95% Confidence Interval Lower Bound	95% Confidence Interval Upper Bound
B	C10	42.975	1.086	40.795	45.154
	C11	24.863	1.008	22.839	26.887
D	C10	67.417	1.008	65.393	69.441
	C11	54.514	1.008	52.491	56.538

11. selection * scissor

Dependent Variable: mutation

selection	scissor	Mean	Std. Error	95% Confidence Interval Lower Bound	Upper Bound
S	C10	82.807	1.008	80.783	84.831
	C11	63.320	1.008	61.296	65.344
Un	C10	27.585	1.086	25.405	29.764
	C11	16.058	1.008	14.034	18.081

12. ruler * source * selection

Dependent Variable: mutation

ruler	source	selection	Mean	Std. Error	95% Confidence Interval Lower Bound	Upper Bound
AUTH	B	S	45.860	1.337	43.176	48.544
		Un	14.710	1.422	11.855	17.565
	D	S	95.653	1.337	92.970	98.337
		Un	21.943	1.337	19.259	24.627
SURR	B	S	51.940	1.337	49.256	54.624
		Un	23.166	1.422	20.311	26.021
	D	S	98.800	1.337	96.116	101.484
		Un	27.466	1.337	24.782	30.150

13. ruler * source * scissor

Dependent Variable: mutation

ruler	source	scissor	Mean	Std. Error	95% Confidence Interval Lower Bound	Upper Bound
AUTH	B	C10	39.388	1.422	36.533	42.243
		C11	21.182	1.337	18.499	23.866
	D	C10	65.296	1.337	62.612	67.980
		C11	52.300	1.337	49.616	54.984
SURR	B	C10	46.562	1.422	43.707	49.417
		C11	28.544	1.337	25.860	31.228
	D	C10	69.538	1.337	66.854	72.221
		C11	56.729	1.337	54.045	59.413

14. ruler * selection * scissor

Dependent Variable: mutation

ruler	selection	scissor	Mean	Std. Error	95% Confidence Interval Lower Bound	95% Confidence Interval Upper Bound
AUTH	S	C10	80.547	1.337	77.863	83.231
		C11	60.967	1.337	58.283	63.651
	Un	C10	24.137	1.422	21.282	26.992
		C11	12.516	1.337	9.832	15.200
SURR	S	C10	85.067	1.337	82.383	87.751
		C11	65.673	1.337	62.989	68.357
	Un	C10	31.033	1.422	28.178	33.888
		C11	19.599	1.337	16.915	22.283

15. source * selection * scissor

Dependent Variable: mutation

source	selection	scissor	Mean	Std. Error	95% Confidence Interval Lower Bound	95% Confidence Interval Upper Bound
B	S	C10	59.946	1.337	57.261	62.630
		C11	37.854	1.337	35.170	40.539
	Un	C10	26.004	1.509	22.975	29.033
		C11	11.872	1.337	9.187	14.557
D	S	C10	105.668	1.337	102.983	108.353
		C11	88.786	1.337	86.101	91.470
	Un	C10	29.166	1.337	26.481	31.850
		C11	20.243	1.337	17.558	22.928

16. ruler * source * selection * scissor

Dependent Variable: mutation

ruler	source	selection	scissor	Mean	Std. Error	95% Confidence Interval Lower Bound	95% Confidence Interval Upper Bound
AUTH	B	S	C10	56.953	1.685	53.569	60.337
			C11	34.768	1.675	31.406	38.130
		Un	C10	21.823	1.854	18.101	25.545
			C11	7.597	1.685	4.214	10.981
	D	S	C10	104.141	1.675	100.779	107.503
			C11	87.166	1.685	83.782	90.549
		Un	C10	26.451	1.685	23.067	29.835
			C11	17.435	1.675	14.073	20.797
SURR	B	S	C10	62.939	1.685	59.555	66.322
			C11	40.941	1.675	37.579	44.303
		Un	C10	30.185	1.854	26.463	33.907
			C11	16.147	1.685	12.763	19.530
	D	S	C10	107.195	1.675	103.833	110.557
			C11	90.406	1.685	87.022	93.790
		Un	C10	31.881	1.685	28.497	35.264
			C11	23.051	1.675	19.689	26.413

Profile Plots

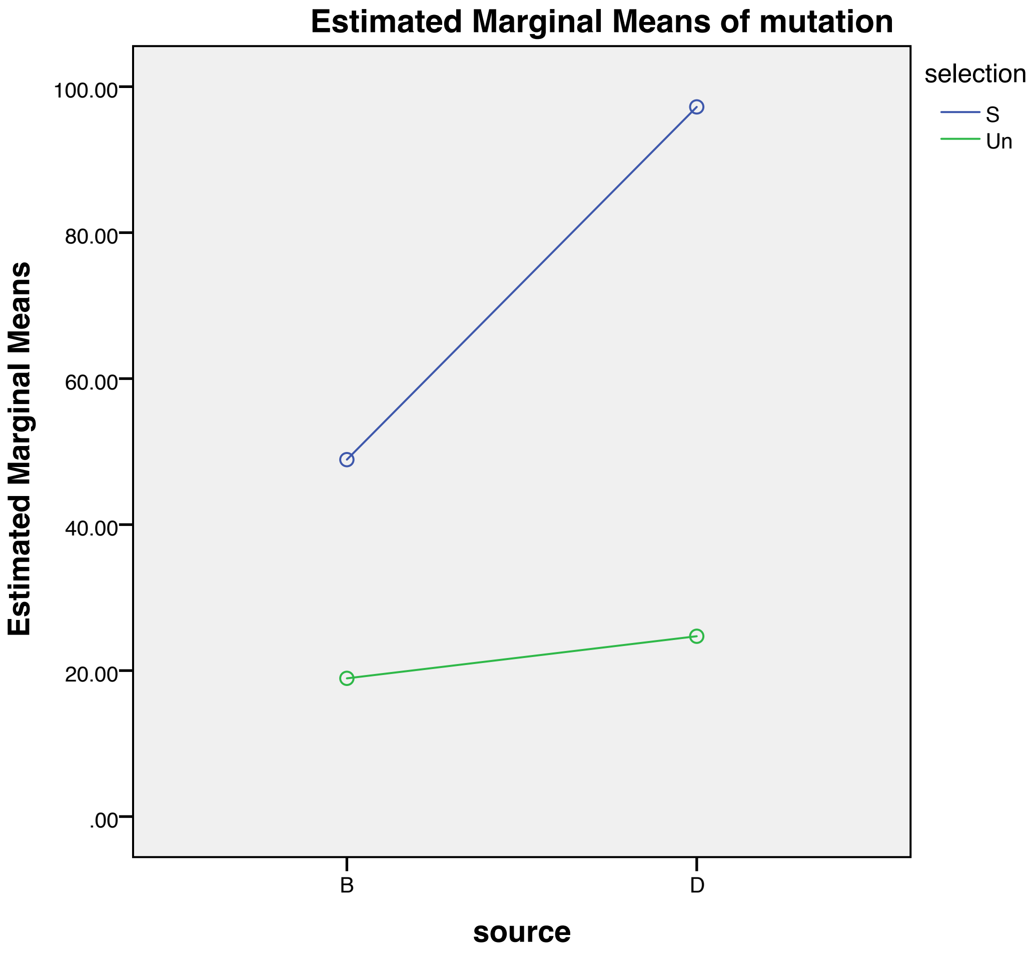

Estimated Marginal Means of mutation
Estimated Marginal Means
100.00
80.00
60.00
40.00
20.00
.00
B
D
source
selection
S
Un

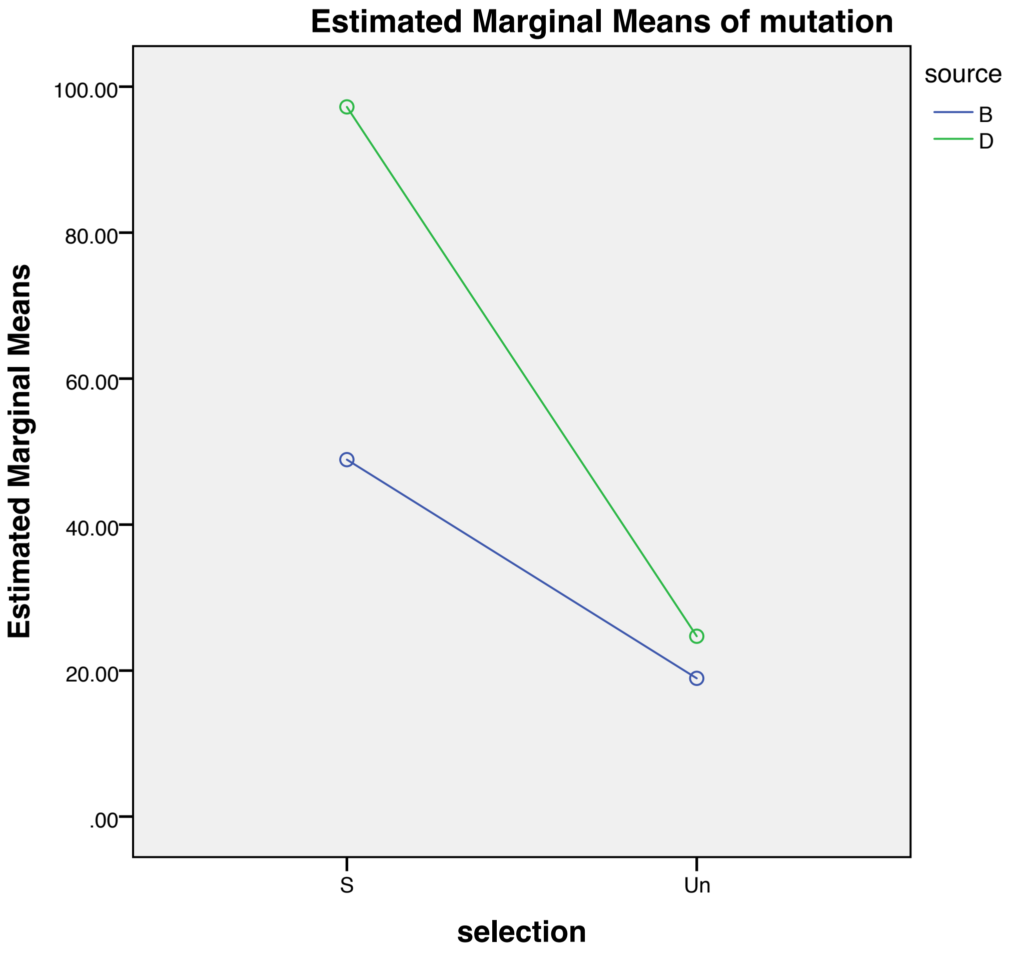

Estimated Marginal Means of mutation
Estimated Marginal Means
100.00
80.00
60.00
40.00
20.00
.00
S
Un
selection
source
B
D

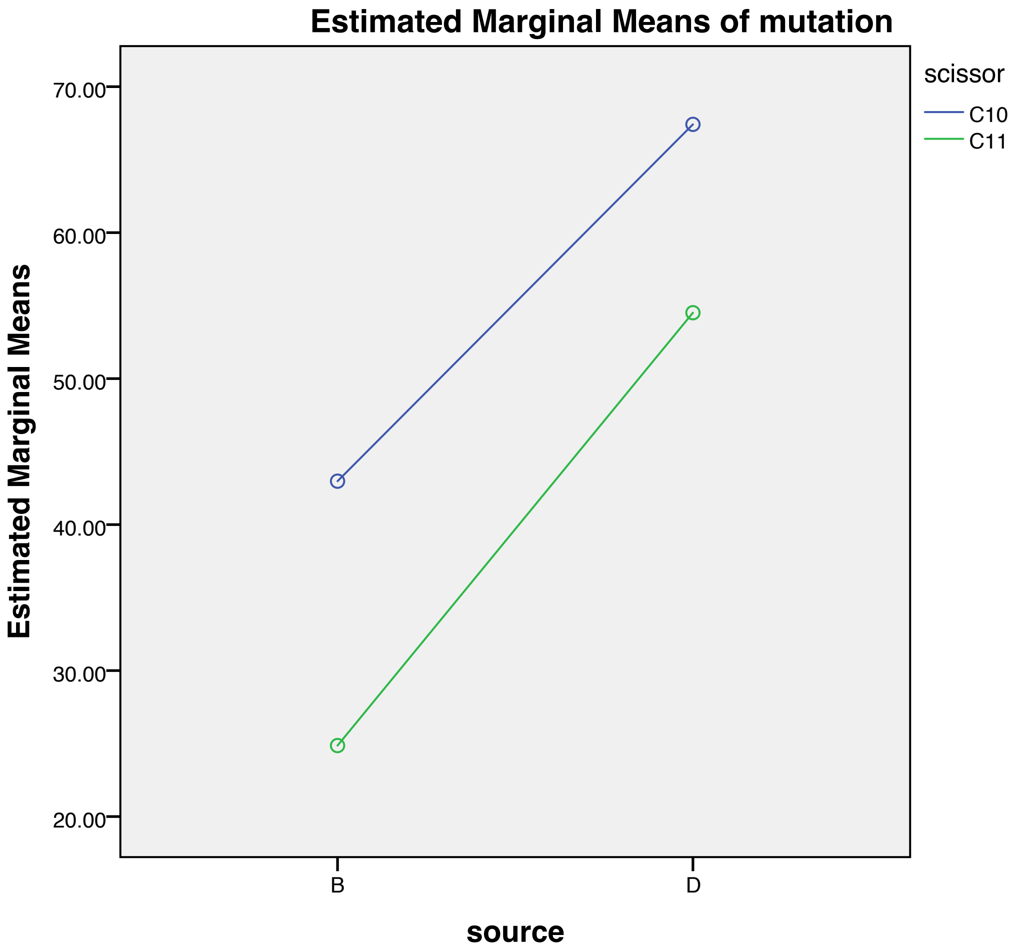

Estimated Marginal Means of mutation
Estimated Marginal Means
70.00
60.00
50.00
40.00
30.00
20.00
B
D
source
scissor
C10
C11

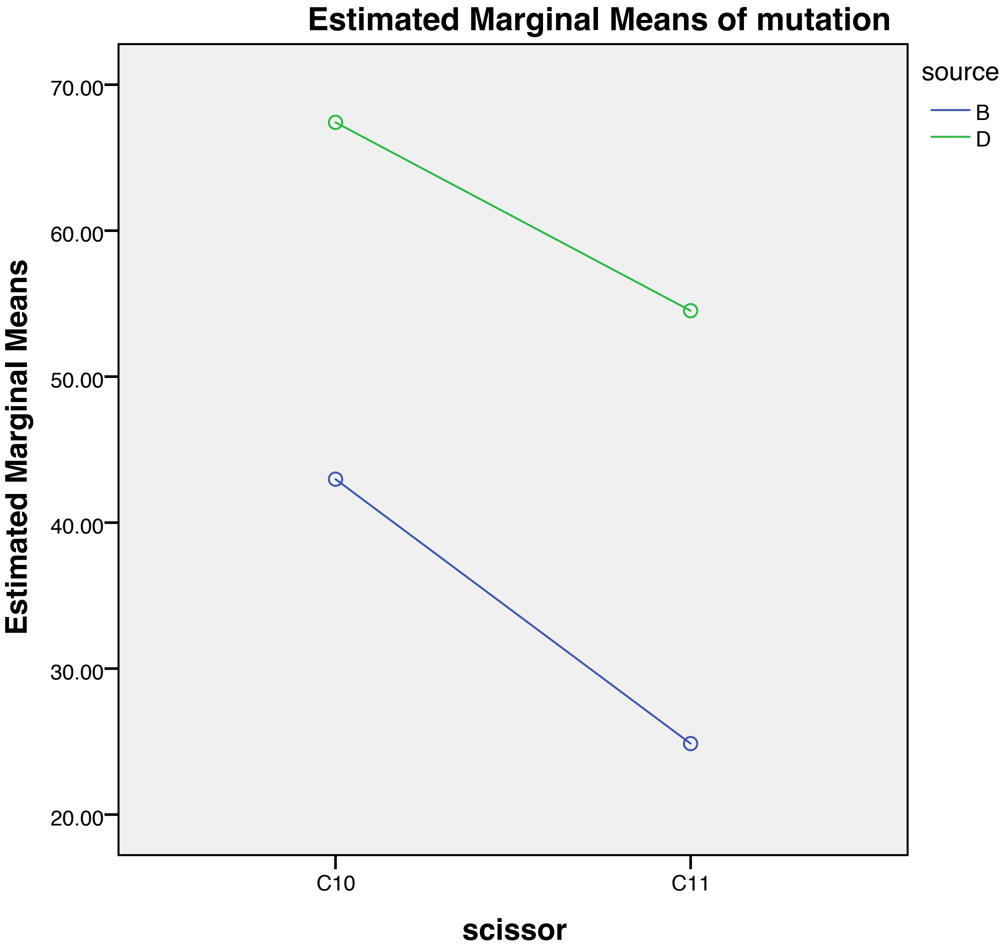
Estimated Marginal Means of mutation
Estimated Marginal Means
70.00
60.00
50.00
40.00
30.00
20.00
C10
C11
scissor
source
B
D

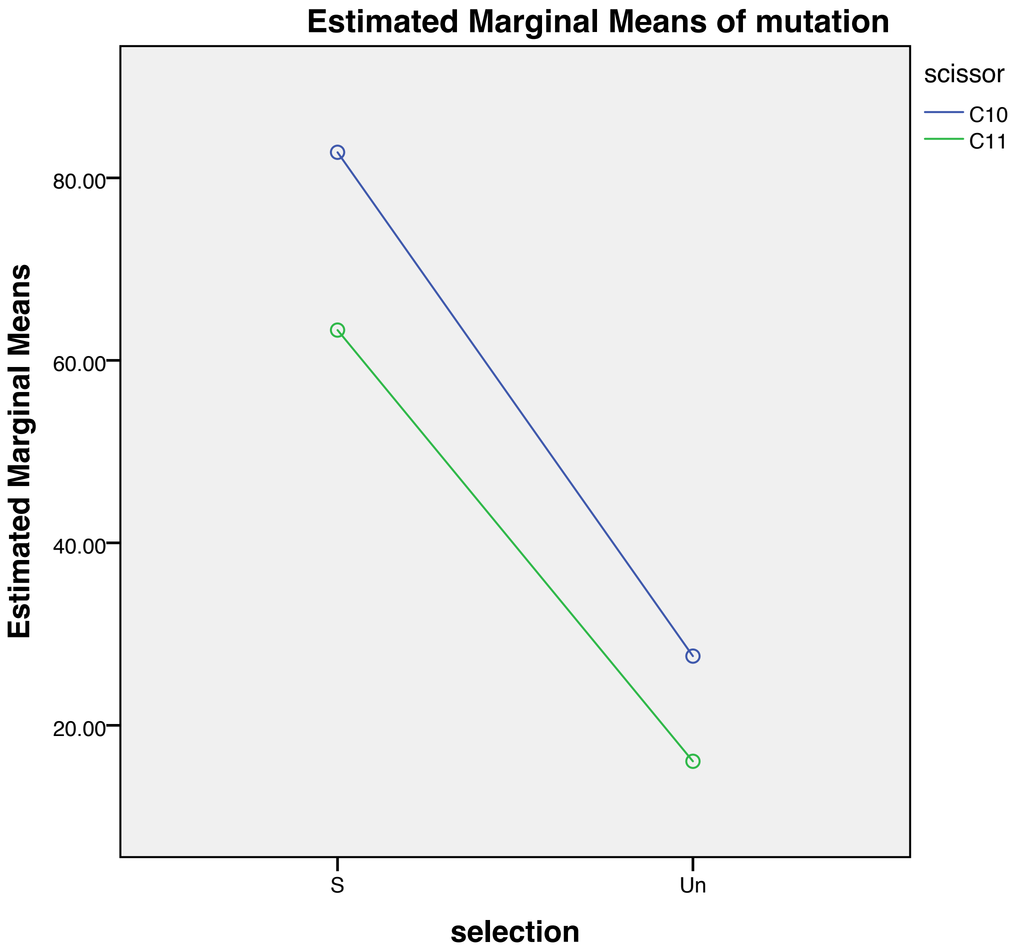
Estimated Marginal Means of mutation
scissor
C10
C11
80.00
60.00
40.00
20.00
Estimated Marginal Means
S
Un
selection

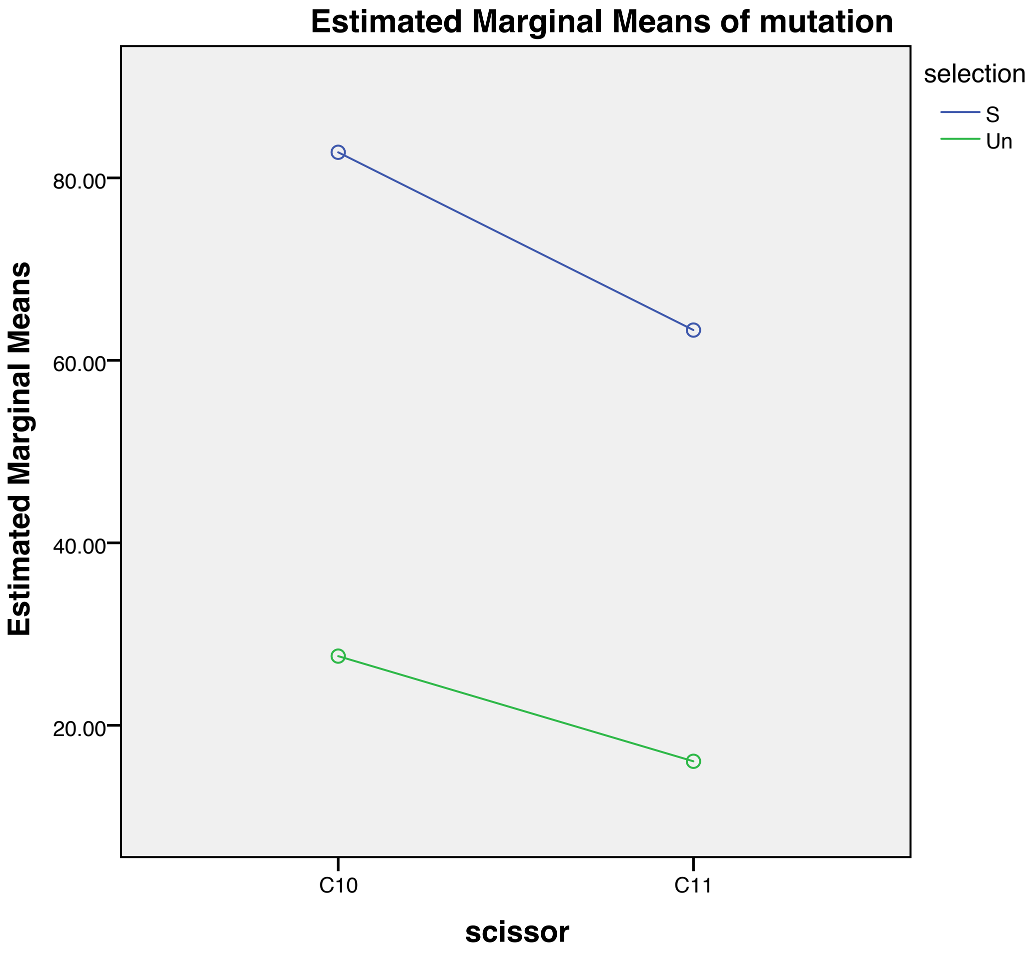
Estimated Marginal Means of mutation
selection
S
Un
80.00
60.00
40.00
20.00
Estimated Marginal Means
C10
C11
scissor
