# Supplementary material for: Non-Homologous End Joining and Homology Directed DNA Repair Frequency of Double-Stranded Breaks Introduced by Genome Editing Reagents
Source: PLoS One. 2017 Jan 17;12(1):e0169931. doi: 10.1371/journal.pone.0169931 (PMC5241150; doi:10.1371/journal.pone.0169931)
Supplement: S1 Appendix — DataFile.xlsx contains the data used for generation of figures and tables and also t-tests on the data. ANOVA analysis reports for Fig 4 and Fig 5 are in ANOVAFig4.pdf and ANOVAFig5.pdf. The NGS.Zip file contains next generation Fasta sequence files (NGSfastafiles.zip), the python module used for NGS analysis (ngsAnalysis_v1.0.py) and output files of NGS analysis (ngsAnalysisDataOutputFiles.zip). S1 Appendix also contains the gel image files (Gels.zip) showing gels with entire lanes of those shown cropped in Fig 13 and original and ‘inverted’ gels for S6 Fig. (ZIP) [file pone.0169931.s001.zip › ANOVAFig5.pdf]

## Tests of Between-Subjects Effects

Dependent Variable: dv

| Source | Type III Sum of Squares | df | Mean Square | F | Sig. |
|---|---|---|---|---|---|
| Corrected Model | 4.339[a] | 9 | .482 | 86.841 | .000 |
| Intercept | 10.376 | 1 | 10.376 | 1868.996 | .000 |
| selected | .792 | 1 | .792 | 142.626 | .000 |
| source | .611 | 1 | .611 | 110.138 | .000 |
| ruler | 1.422 | 2 | .711 | 128.033 | .000 |
| selected * source | .057 | 1 | .057 | 10.273 | .003 |
| selected * ruler | .082 | 2 | .041 | 7.387 | .002 |
| source * ruler | .010 | 1 | .010 | 1.870 | .182 |
| selected * source * ruler | 2.826E-5 | 1 | 2.826E-5 | .005 | .944 |
| Error | .167 | 30 | .006 | | |
| Total | 14.977 | 40 | | | |
| Corrected Total | 4.505 | 39 | | | |

a. R Squared = .963 (Adjusted R Squared = .952)

selected : Puromycin-selected vs unselected samples
source: pBackbone vs pDonor-F8 transfected samples
ruler: F8S2 clone 10 vs clone 11 transfected samples
