# Supplementary figures and images for: Non-Homologous End Joining and Homology Directed DNA Repair Frequency of Double-Stranded Breaks Introduced by Genome Editing Reagents
Source: PLoS One. 2017 Jan 17;12(1):e0169931. doi: 10.1371/journal.pone.0169931 (PMC5241150; doi:10.1371/journal.pone.0169931)

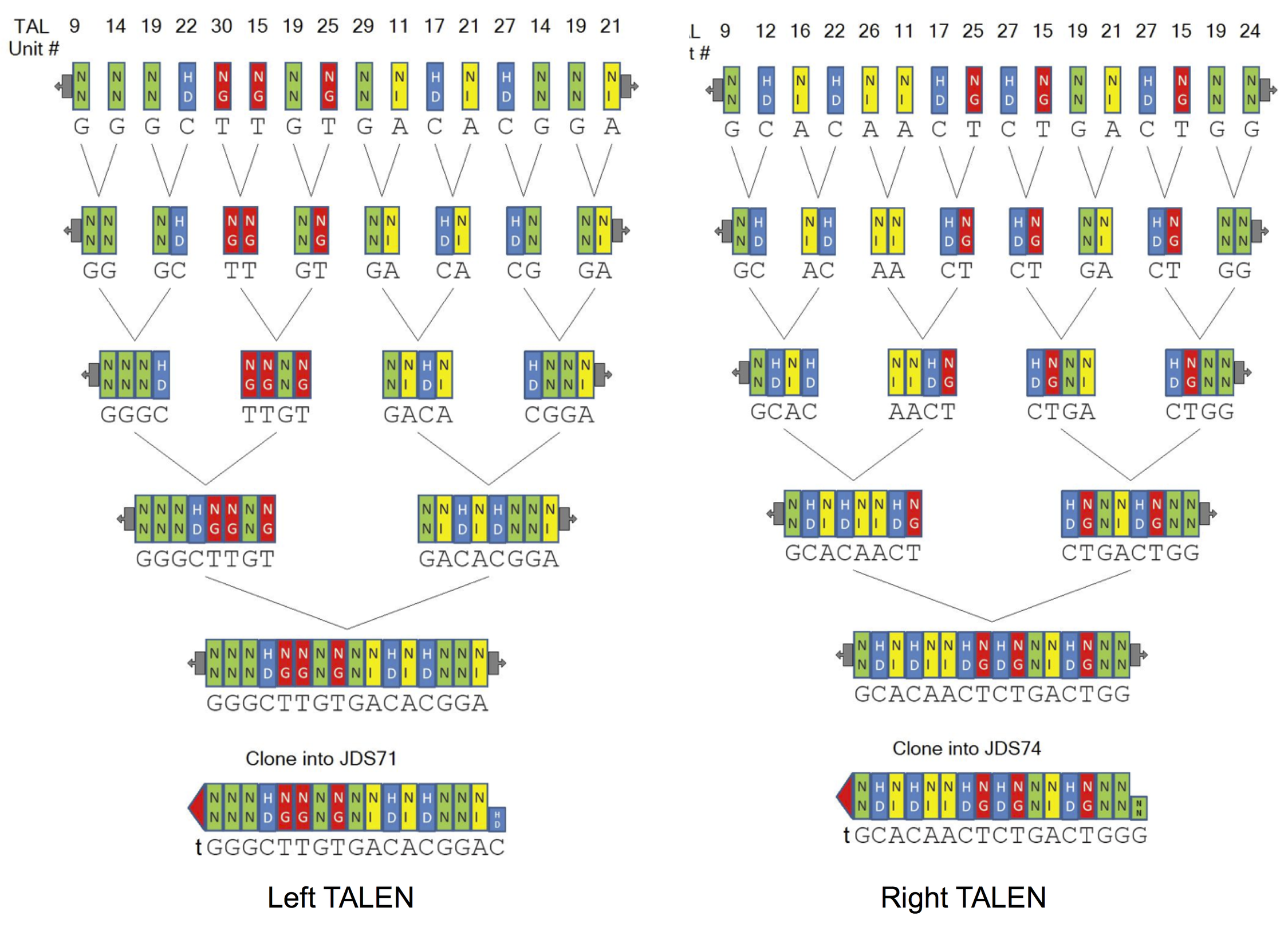

Supplement: S1 Fig — The top row of rectangles depicts the TALE repeat units and the repeat variable di-residue (RVD) with the associated nucleotide specificity. Adjacent repeats were ligated in four sequential steps as shown to create the left and right TALE sequences that were then cloned into the eukaryotic expression plasmid vectors JDS71 and JDS74, respectively. Additional details of cloning (e.g., restriction enzymes used for directional cloning and assembly of repeats) are available in the protocol accompanying the Addgene TALEN Assembly Kit. (TIF) [file pone.0169931.s002.tif]

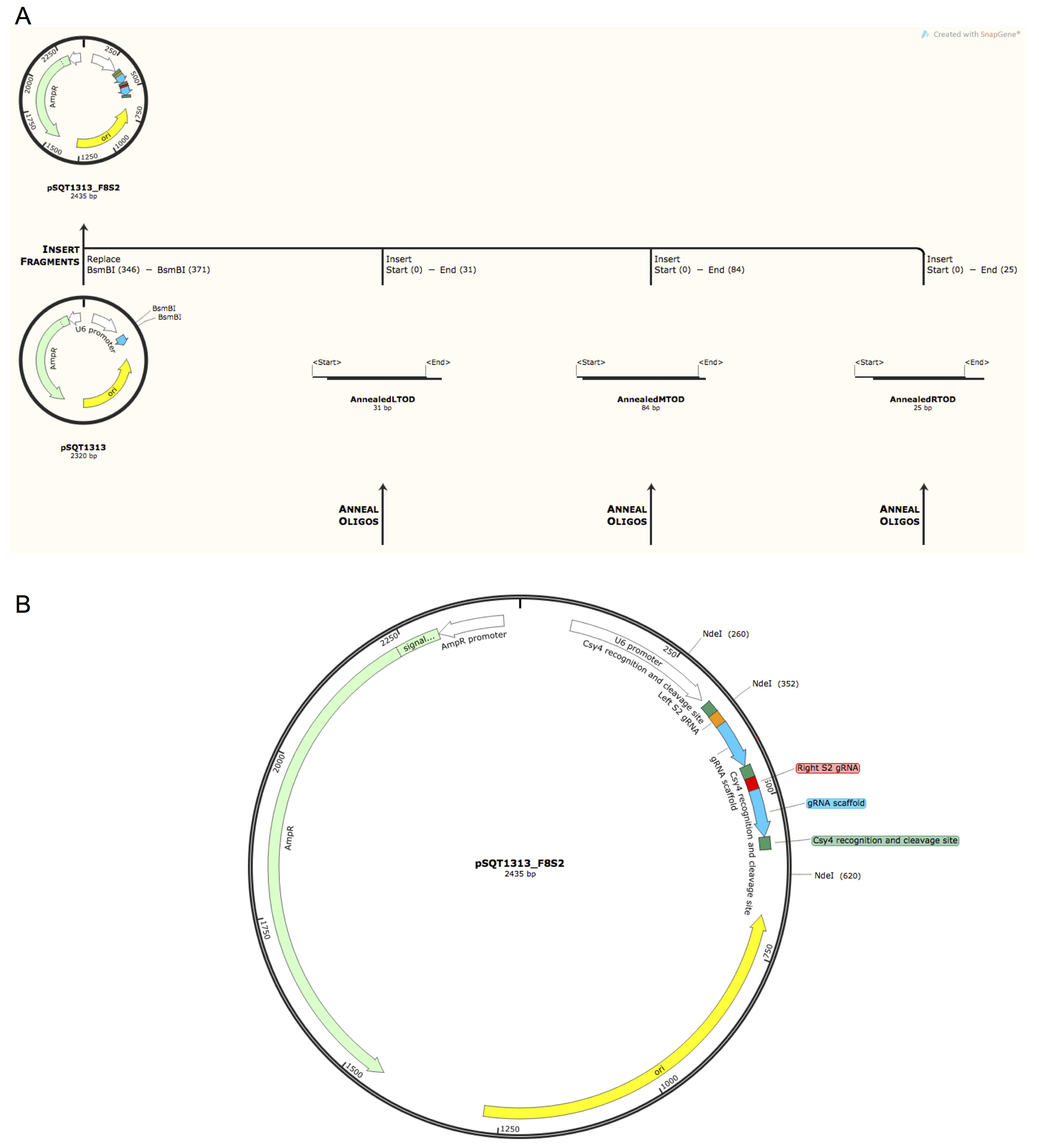

Supplement: S2 Fig — (A) Cloning steps for creation of plasmid pSQT1313_F8S2. The dimeric gRNA expression construct was created by annealing oligos to generate left, middle and right oligo duplexes (AnnealedLTOD, AnnealedMTOD and AnnealedRTOD) specific for F8 S2 locus (S1 Table). These were then combined and ligated into BsmB1 digested pSQT1313 to generate pSQT1313_F8S2. An identical strategy was used for generation of dimeric gRNA expression constructs for targeting F8-S1 (pSQT1313_F8S1) and F8-S3 (pSQT1313_F8S3) sites. Please refer to Material and Methods for additional details. (B) Plasmid map of pSQT1313_F8S2. The right and left S2 gRNAs, the Csy4 recognition and cleavage sites, and other salient features are shown in the dimeric gRNA expression construct. (TIF) [file pone.0169931.s003.tif]

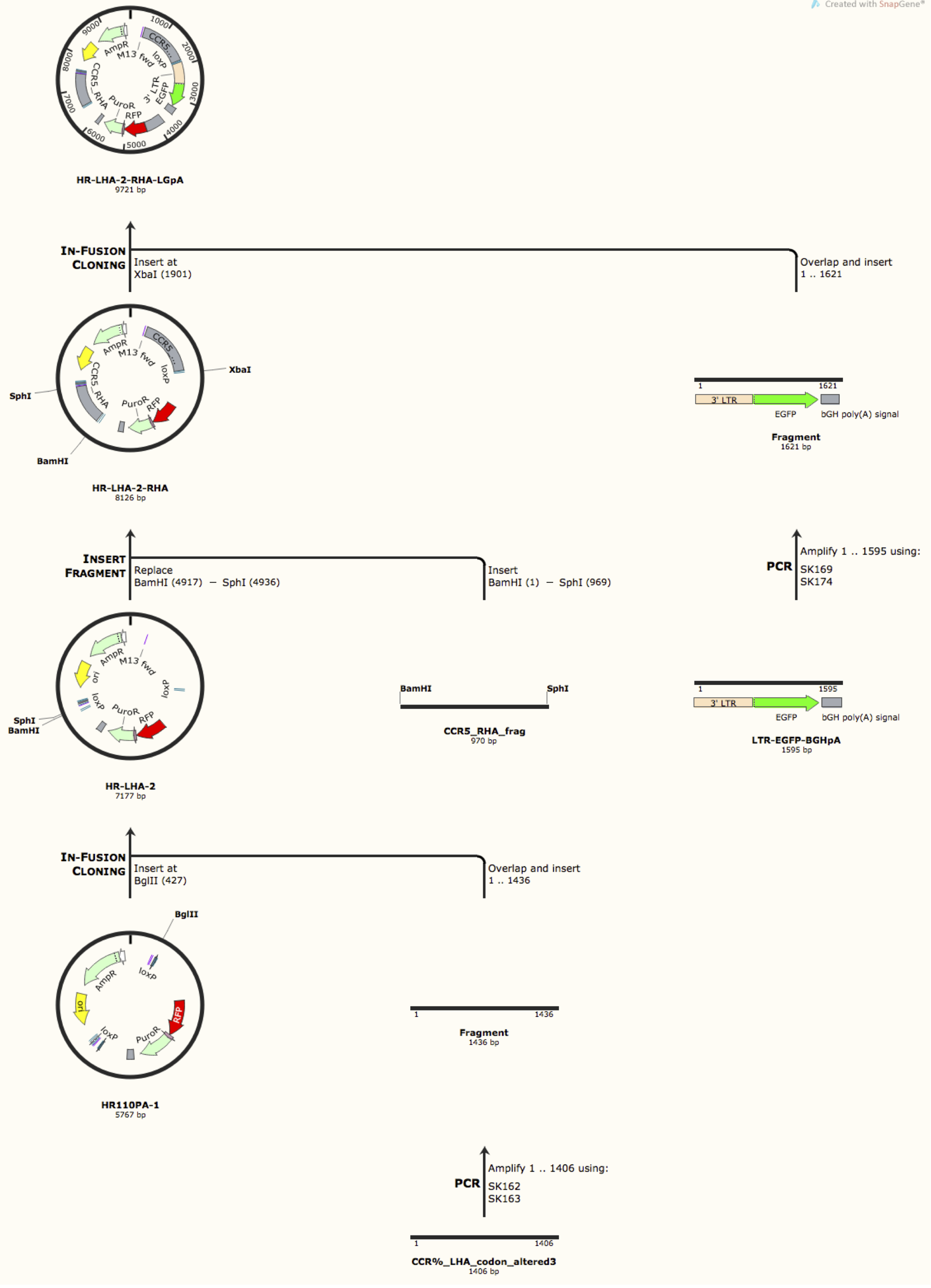

Supplement: S3 Fig — The donor plasmid for homologous recombination at the CCR5 target site was generated as outlined. The left and right homology arms (LHA and RHA) were generated by PCR of genomic DNA using primers listed in S2 Table. These fragments were inserted using either In-Fusion (i.e., ligation-independent) cloning (LHA) or via the introduced restriction enzyme sites (RHA). A second internal expression cassette consisting of HIV-1 LTR promoter driving EGFP and bGH poly(A) was generated by SOE-PCR and inserted at the XbaI site by ligation-independent cloning. Please refer to Material and Methods for details. (TIF) [file pone.0169931.s004.tif]

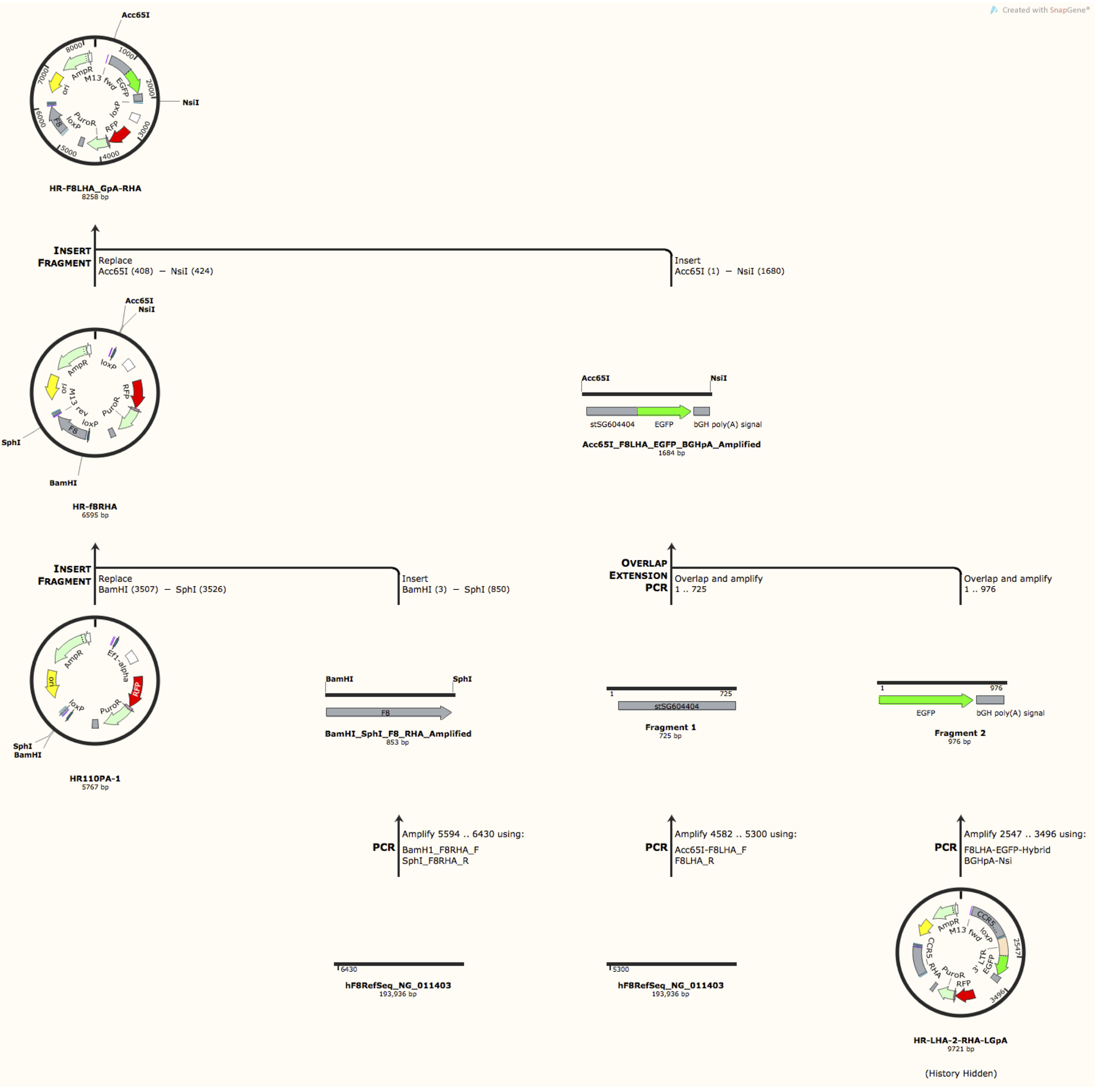

Supplement: S4 Fig — The donor plasmid was generated using the steps depicted in this flow chart. Briefly, it consists of amplifying the right homology arm (RHA) from genomic DNA and inserting it into the multi-cloning site between BamHI and SphI restriction enzyme sites in pBackbone. The left homology arm containing the F8 first coding exon was amplified from human gDNA and fused in frame with EGFP coding sequence and terminated with bGH poly(A) signal sequence using splicing by overlap amplification. This fragment was introduced into the multiple cloning site for the left homology arm (LHA) in pBackbone. The oligonucleotide primers used for generating pDonor-F8 cloning are listed in S3 Table. Please refer to Material and Methods for additional details. (TIF) [file pone.0169931.s005.tif]

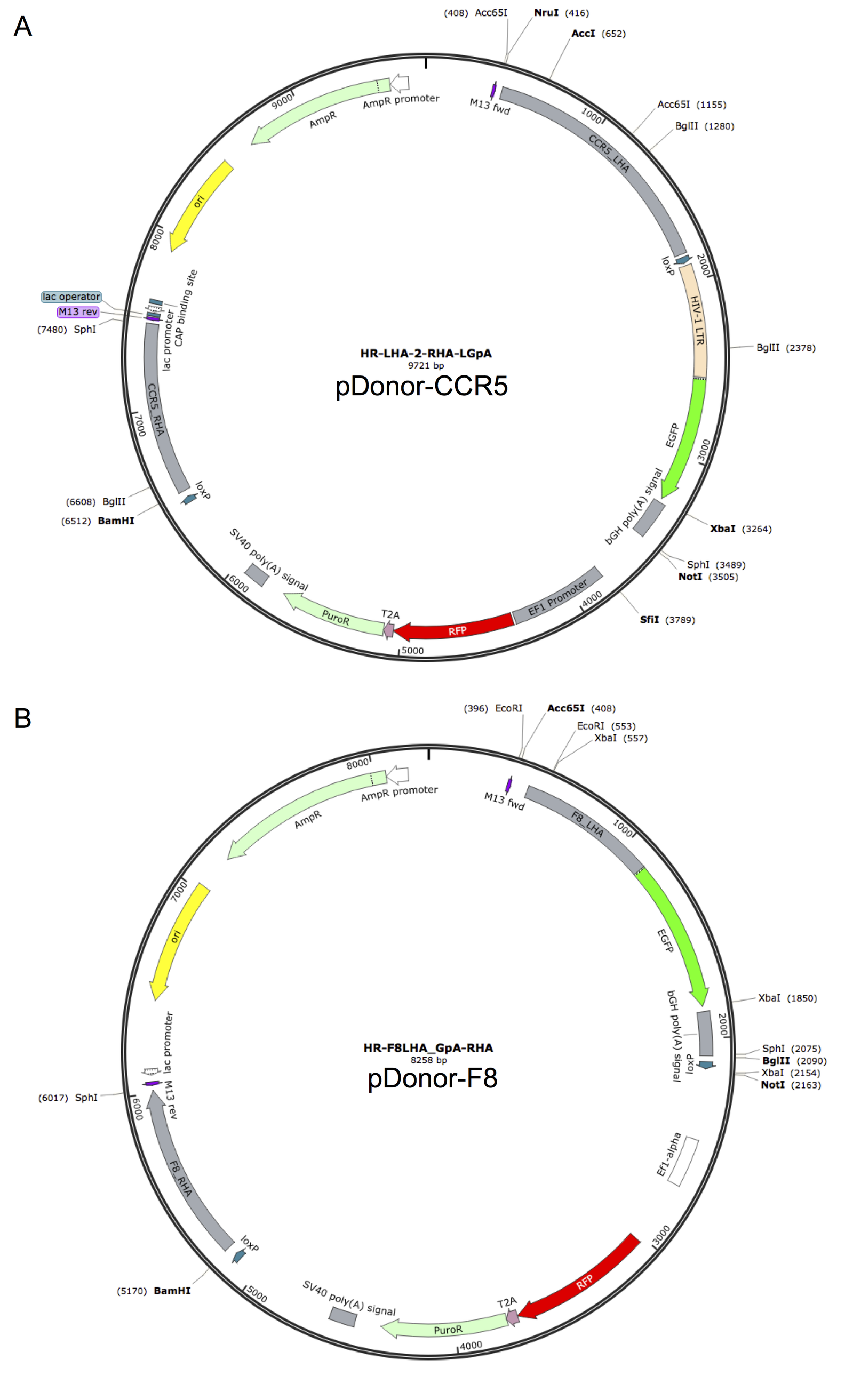

Supplement: S5 Fig — (A) pDonor-CCR5 (HR-LHA-2-RHA-LGpA). This plasmid contains the left (CCR5_LHA) and right (CCR5_RHA) homology arms to effect site specific recombination across the TALEN target cut site, an additional internal expression cassette consisting of the HIV-1 long terminal repeat (HIV-1 LTR) promoter driving enhanced green fluorescent protein (EGFP) and the bovine growth hormone (bGH) polyA site. (B) Plasmid map of pDonor-F8 (HR-F8LHA_GpA-RHA). This plasmid contains the left (F8_LHA) and right (F8_RHA) homology arms to effect site specific recombination across the F8 S1, S2 or S3 cut sites, EGFP in frame with F8 coding exon 1 and the bGH polyA sequence. Both donor plasmids contain an expression cassette consisting of the elongation factor-1 alpha (EF1-alpha) promoter driving a fusion gene composed of red fluorescent protein (RFP), self-cleaving picornavirus protease (T2A) and puromycin N-acetyl-transferase (PuroR) and terminated with the simian virus 40 (SV40) polyA signal. Other elements required for replication (ori) and selection (AmpR) in bacteria are also shown. (TIF) [file pone.0169931.s006.tif]

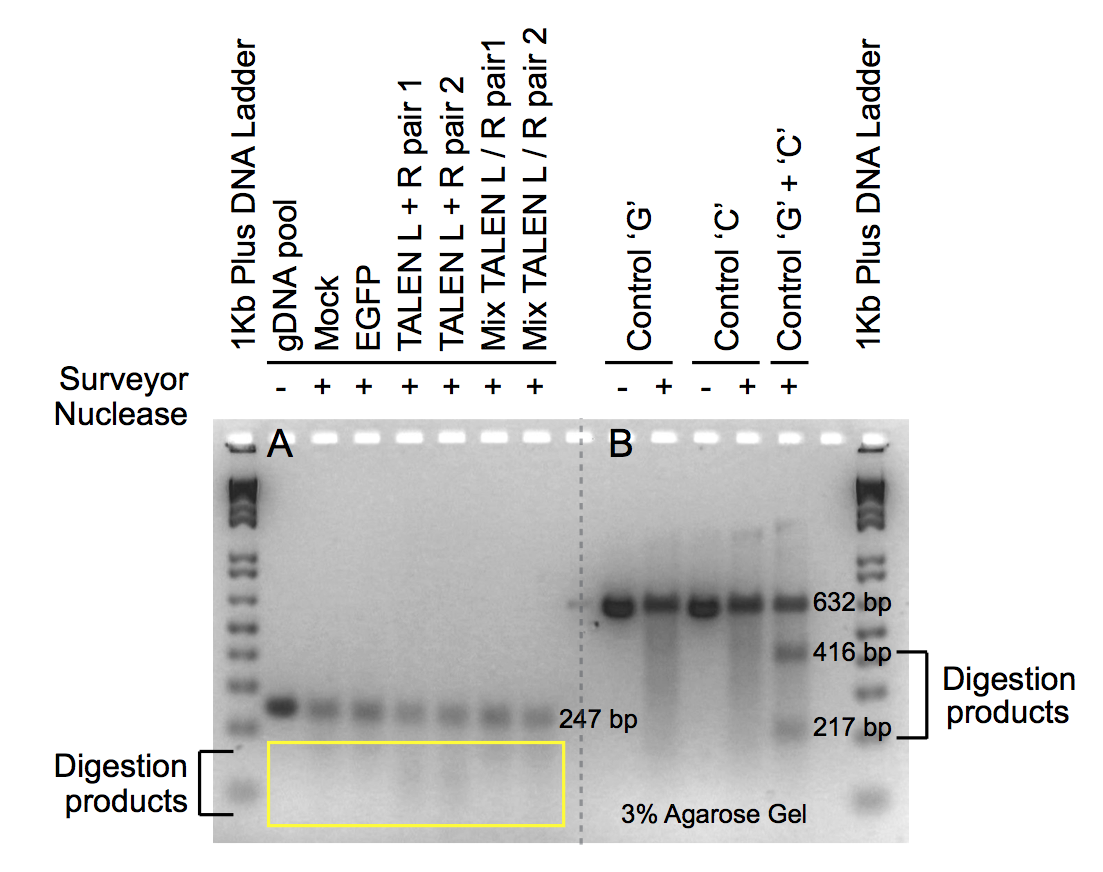

Supplement: S6 Fig — A) HEK293T cells were transfected mock transfected or transfected with an EGFP encoding plasmid (EGFP) or left (L) TALEN, right (R) TALEN or both. Two pairs of left and right TALENs were tested. gDNA from each of the transfections were subjected to PCR using primer pair SK214 (5’-TGCTGTTCTATTTTCCAGCAA-3’) and SK215 (5’-CAGATGCCAAATAAATGGATGA-3’). These primers amplify a 247 bp product between nt 8728 and nt 8974 of CCR5 RefSeq (GenBank accession no. NG_012637). The PCR products were then digested with Surveyor Nuclease (Transgenomics Inc, USA) as per the recommended protocol and resolved on a 3% agarose gel. The region showing a smear of digestion products is enclosed within the yellow rectangle. B)‘G’ and ‘C’ controls from the Surveyor Nuclease kit produce a PCR product of size 632 bp. Upon mixing and reannealing, Surveyor nuclease digestion yields products of size 416 bp and 217 bp. (TIFF) [file pone.0169931.s007.tiff]
