# Supplementary material for: Non-Homologous End Joining and Homology Directed DNA Repair Frequency of Double-Stranded Breaks Introduced by Genome Editing Reagents
Source: PLoS One. 2017 Jan 17;12(1):e0169931. doi: 10.1371/journal.pone.0169931 (PMC5241150; doi:10.1371/journal.pone.0169931)
Supplement: S1 Table — (DOCX) [file pone.0169931.s008.docx]

| **S1 Table. Oligonucleotide primers used for creation of dimeric-gRNA encoding plasmids targeting FVIII intron 1 sites S1, S2, and S3.** | | |
| --- | --- | --- |
| **Oligonucleotide** | **Description** | **Sequence (5’-3’)** |
| LTOD_s1_F | S1, Left OD*, S | GCAGACTTACATCCCCACAATCCGTTTTAG |
| LTOD_s1_R | S1, Left OD, AS | AGCTCTAAAACGGATTGTGGGGATGTAAGT |
| RTOD_s1_F | S1, Right OD, S | GGCAGTGCAGACATCGGGTTAGGA |
| RTOD_s1_R | S1, Right OD, AS | AAACTCCTAACCCGATGTCTGCAC |
| LTOD_s2_F | S2, Left OD, S | GCAGCATATGACGAAGAGAAGCAAGTTTTAG |
| LTOD_s2_R | S2, Left OD, AS | AGCTCTAAAACTTGCTTCTCTTCGTCATATG |
| RTOD_s2_F | S2, Right OD, S | GGCAGTAGAGACTGGAGTAGAGGGA |
| RTOD_s2_R | S2, Right OD, AS | AAACTCCCTCTACTCCAGTCTCTAC |
| LTOD_s3_F | S3, Left OD, S | GCAGATTATCCTTACACCCAAAGTGTTTTAG |
| LTOD_s3_R | S3, Left OD, AS | AGCTCTAAAACACTTTGGGTGTAAGGATAAT |
| RTOD_s3_F | S3, Right OD, S | GGCAGAGAAGCTTTTCGTGTTTTGG |
| RTOD_s3_R | S3, Right OD, AS | AAACCCAAAACACGAAAAGCTTCTC |
| Middle_Oligo_F | Middle oligo, S, P^a^ | AGCTAGAAATAGCAAGTTAAAATAAGGCTAGTCCGTTAT**C**AACTTGAAAAAGTGGCACCGAGTCGGTGCGTTCACTGCCGTATA |
| Middle_Oligo_R | Middle oligo, AS, P^a^ | TGCCTATACGGCAGTGAACGCACCGACTCGGTGCCACTTTTTCAAGTTGATAACGGACTAGCCTTATTTTAACTTGCTATTTCT |
| gRNA Sequencing primer |  | AGGGTTATTGTCTCATGAGCGG |

^a^OD: Oligo duplex; P: phosphorylated oligonucleotide, S: sense primer, AS: antisense primer; S1, S2 and S3 refer to target site in intron 1 of F8 RefSeq (NG_011403)
