# Supplementary material for: Non-Homologous End Joining and Homology Directed DNA Repair Frequency of Double-Stranded Breaks Introduced by Genome Editing Reagents
Source: PLoS One. 2017 Jan 17;12(1):e0169931. doi: 10.1371/journal.pone.0169931 (PMC5241150; doi:10.1371/journal.pone.0169931)
Supplement: S2 Table — (DOCX) [file pone.0169931.s009.docx]

| **S2 Table. Oligonucleotide primers used for creation of pDonor-CCR5** | | |
| --- | --- | --- |
| **Primer** | **Description^a^** | **Sequence^b^** |
| SK162 | CCR5-pBackbone hybrid, LHA, S at BglII site | CGAATGCATCAGATCCTGAGCTGCACCATGCTTGA |
| SK163 | CCR5-pBackbone hybrid, LHA, AS at BglII site | TTGAGTGGAAAGATCCTATAATCCTACAGATATTTCCTGCTCCCC |
| SK164 | CCR5, RHA, S, with introduced BamHI site | CGGGATCCTGGGGTGGGAGAGGTCTTTT |
| SK165 | CCR5, RHA, AS, with introduced SphI site | ATGCATGCCTGACCCTCACTTCCAACCC |
| SK169 | HIV-1 LTR-pBackbone hybrid at XbaI site S | TATTCAAAAATCTAGTGGAAGGGCTAATTCACTCC |
| SK170 | HIV-1 LTR, AS | TGCTAGAGATTTTCCACACT |
| SK171 | HIV-1 LTR-EGFP hybrid, S | GGAAAATCTCTAGCACGCCACCATGGTGAGCAAGG |
| SK172 | EGFP, AS | ACTTGTACAGCTCGTCCATG |
| SK173 | EGFP-BGHpA hybrid, S | GAGCTGTACAAGTAACTAGAGCTCGCTGATCAGCC |
| SK174 | BGHpA-pBackbone hybrid, AS at XbaI site | GCGGCCGCTGTCTAGTCCCCAGCATGCCTGCTATT |

^a^LHA: Left homology arm, RHA: Right homology arm, S: Sense, AS: Antisense; EGFP: Enhanced green fluorescent protein, BGHpA: Bovine growth hormone poly A, HIV-1: Human immunodeficiency virus type 1; LTR: Long terminal repeat.

^b^The oligonucleotide sequences for CCR5 originated from RefSeqGene NG_012637, HIV-1 LTR originated from pNL4-3 (GenBank Accession number M19921), nt 9076 to nt 9709; EGFP sequence originated from pEGFP-N1 (Clontech), nt 679 to nt 1398; bGHpA sequence originated from pCDNA3 (Invitrogen), nt 1042 to nt 1249.
