# Supplementary material for: Non-Homologous End Joining and Homology Directed DNA Repair Frequency of Double-Stranded Breaks Introduced by Genome Editing Reagents
Source: PLoS One. 2017 Jan 17;12(1):e0169931. doi: 10.1371/journal.pone.0169931 (PMC5241150; doi:10.1371/journal.pone.0169931)
Supplement: S3 Table — (DOCX) [file pone.0169931.s010.docx]

| **S3 Table. Oligonucleotide primers used for creating pDonor-F8** | | |
| --- | --- | --- |
| **Primer** | **Description** | **Sequence*** |
| BamHI_F8RHA_F | RHA with BamHI site, S | cgggatccGGGAGGGTGAAGGGACAAAG |
| SphiI_F8RHA_R | RHA with SphI site, AS | ATGCATGCGATGTGGAAAGAGGGTGGGG |
| Acc65I-F8LHA_F | LHA with Acc65I site, S | ggtaccACAATAGTTGCCTAACCTCATGT |
| F8_LHA_R | LHA, AS | CAGCTCACCGAGATCACTT |
| F8LHA-EGFP-Hybrid_F | LHA-EGFP hybrid, S | gtgatctcggtgagctgATGGTGAGCAAGGGCGAGG |
| SK173 | EGFP-BGHpA hybrid, S | GAGCTGTACAAGTAACTAGAGCTCGCTGATCAGCC |
| BGHpA_NsiI_R | BGHpA with NsiI site, AS | ctgatgcatTCCCCAGCATGCCTGCTATT |

S: Sense primer, AS: Antisense primer, RHA: Right homology arm, LHA: Left homology arm, EGFP: Enhanced green fluorescent protein, BGHpA: Bovine growth hormone poly A

*The F8 sequences were derived from GenBank Accession Number NG_011403
